# Diversity and Paleodemography of the Addax (*Addax nasomaculatus*), a Saharan Antelope on the Verge of Extinction

**DOI:** 10.3390/genes12081236

**Published:** 2021-08-11

**Authors:** Elisabeth Hempel, Michael V. Westbury, José H. Grau, Alexandra Trinks, Johanna L. A. Paijmans, Sergei Kliver, Axel Barlow, Frieder Mayer, Johannes Müller, Lei Chen, Klaus-Peter Koepfli, Michael Hofreiter, Faysal Bibi

**Affiliations:** 1 Evolutionary Adaptive Genomics, Institute of Biochemistry and Biology, Faculty of Science, University of Potsdam, Karl-Liebknecht-Straße 24-25, 14476 Potsdam, Germany; jh.grau.jipoulou@gmail.com (J.H.G.); michael.hofreiter@uni-potsdam.de (M.H.); 2 Museum für Naturkunde, Berlin, Leibniz Institute for Evolution and Biodiversity Science, Invalidenstraße 43, 10115 Berlin, Germany; Frieder.Mayer@mfn.berlin (F.M.); Johannes.Mueller@mfn.berlin (J.M.); Faysal.Bibi@mfn.berlin (F.B.); 3 Section for Evolutionary Genomics, The GLOBE Institute, University of Copenhagen, Øster Voldgade 5-7, 1350 Copenhagen, Denmark; m.westbury@sund.ku.dk; 4 Institute of Pathology, Charité–Universitätsmedizin Berlin, Charitéplatz 1, 10117 Berlin, Germany; alexandra.trinks@bih-charite.de; 5 Department of Zoology, University of Cambridge, Downing Street, Cambridge CB2 3EJ, UK; paijmans.jla@gmail.com; 6 Institute of Molecular and Cellular Biology SB RAS, 8/2 Acad. Lavrentiev Ave, 630090 Novosibirsk, Russia; mahajrod@gmail.com; 7 School of Science and Technology, Nottingham Trent University, Clifton Lane, Nottingham NG11 8NS, UK; axel.barlow.ab@gmail.com; 8 School of Ecology and Environment, Northwestern Polytechnical University, Xi’an 710072, China; chenlei@mail.kiz.ac.cn; 9 Smithsonian-Mason School of Conservation, George Mason University, Front Royal, VA 22630, USA; klauspeter.koepfli527@gmail.com; 10 Smithsonian Conservation Biology Institute, Center for Species Survival, National Zoological Park, Front Royal, VA 22630, USA; 11 Computer Technologies Laboratory, ITMO University, 197101 Saint Petersburg, Russia

**Keywords:** *Addax nasomaculatus*, antelope, archival DNA, bovid, conservation, critically endangered, genome assembly, museum collections, PSMC

## Abstract

Since the 19th century, the addax (*Addax nasomaculatus*) has lost approximately 99% of its former range. Along with its close relatives, the blue antelope (*Hippotragus leucophaeus*) and the scimitar-horned oryx (*Oryx dammah*), the addax may be the third large African mammal species to go extinct in the wild in recent times. Despite this, the evolutionary history of this critically endangered species remains virtually unknown. To gain insight into the population history of the addax, we used hybridization capture to generate ten complete mitochondrial genomes from historical samples and assembled a nuclear genome. We found that both mitochondrial and nuclear diversity are low compared to other African bovids. Analysis of mitochondrial genomes revealed a most recent common ancestor ~32 kya (95% CI 11–58 kya) and weak phylogeographic structure, indicating that the addax likely existed as a highly mobile, panmictic population across its Sahelo–Saharan range in the past. PSMC analysis revealed a continuous decline in effective population size since ~2 Ma, with short intermediate increases at ~500 and ~44 kya. Our results suggest that the addax went through a major bottleneck in the Late Pleistocene, remaining at low population size prior to the human disturbances of the last few centuries.

## 1. Introduction

**Figure 1:**
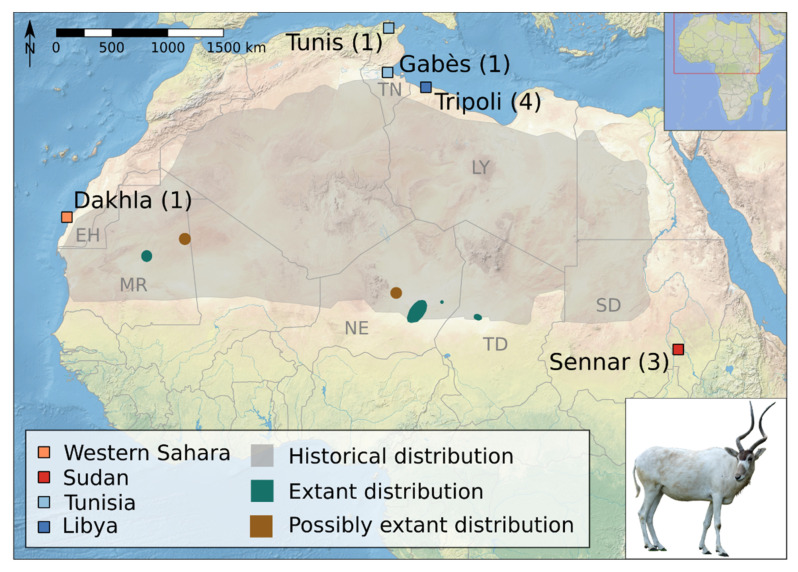
**Map of sample locations**. Historical and (possible) extant distribution of the addax [1,6] and location of historical samples with sample sizes in parenthesis (photo credit: E. Hempel; base map: https://www.naturalearthdata.com, accessed on 18 October 2017, generated in QGIS v2.18 https://www.qgis.org). EH: Western Sahara, LY: Libya, MR: Mauritania, NE: Niger, SD: Sudan, TD: Chad, TN: Tunisia.

Characterizing the population genetics of species on the verge of extinction is of vital importance, both for guiding conservation efforts as well as gathering baseline data that can be used to guide post-extinction restoration from captive populations. One species that exemplifies this dire situation is the addax, *Addax nasomaculatus* (de Blainville, 1816), which is critically endangered and may soon be extinct in the wild [1]. It is highly nomadic, surviving in the hyperarid Sahara by tracking sporadic rainfall that leads to fast vegetation growth [2]. The addax is highly adapted to its desert environment and formerly occurred in great numbers across the entire Sahelo–Saharan region west of the Nile [2,3,4,5]. However, range reductions have resulted in a loss of up to 99% of its historical range [6] (Figure 1). Today, the only documented wild population is in the Réserve Naturelle Nationale du Termit et du Tin Toumma (TTNNR) in eastern Niger, but its status is not well known: The most recent surveys in 2016 and 2017 found only three and six individuals, respectively [1,6,7,8]. A legal re-classification of the status of the TTNNR to make way for oil concessions in 2019 means that the addax’s last wild home is no longer protected [9]. There are sporadic accounts of addax in other parts of Niger, western Chad, and Mauritania [1,4,5,10] (Figure 1), and a few individuals from these populations might have strayed to northern Niger, southern Algeria, and Libya [1,5]. Finally, there are also unconfirmed reports of addax at the Mali–Mauritania border [1,5]. Overall, the global wild population is estimated to be less than 100 with only 30–90 mature individuals [1]. The addax is listed in Appendix I of the Convention on International Trade in Endangered Species of Wild Fauna and Flora (CITES) and the Convention on the Conservation of Migratory Species of Wild Animals (CMS) [1]. In contrast to their meagre numbers in the wild, ~760 individuals are kept in zoos in Europe, North America, Japan, and Australia, ~5000 are found on private ranches in the United States and the Middle East [1,11], and reintroduction programs have brought individuals into national parks or fenced-in areas in Tunisia and Morocco [1,12].

Since the early 20th century, writers have remarked on the many threats facing the addax [2,13]. The greatest among these is unsustainable hunting, particularly since the advent of motor vehicles and modern firearms [3,5,14]. Furthermore, hunting by military personnel accompanying oil and mining exploration appears to have caused massive declines in addax numbers in the TTNNR and adjacent areas [7,14,15,16]. In addition, the extension of pastoralism into the desert has resulted in habitat degradation and competition with domestic livestock [3,4,5,14]. Regional insecurity, droughts and desertification, irresponsible tourism, as well as habitat loss due to oil exploration and exploitation, contribute to the addax’s poor situation [3,5,12,14,17,18,19,20,21].

Despite its highly threatened status in the wild, we know almost nothing about the evolutionary history of the addax. There are only two reports of Pleistocene remains assigned to this species, a maxilla from Algeria described by Balout [22], which is more likely an alcelaphin, and a single deciduous tooth from Morocco described by Thomas [23], which is quite undiagnostic. The addax therefore has no fossil record prior to the Holocene [24,25,26]. The addax is a monotypic member of the bovid tribe Hippotragini and diverged from its sister clade *Oryx* spp. ~3 million years ago based on age estimates using mitochondrial genomes [27]. In addition, desert ecosystems have received less attention in conservation biology compared to other ecosystems and therefore there is a strong need to create baseline biodiversity data [6,28,29,30]. Little is known about the genetic diversity and structure of either past or current populations of the addax and no reference nuclear genome is publicly available. Important questions remain outstanding: Was the demise of the addax a recent (20th century) phenomenon, or were its numbers already low during the Pleistocene? Were addax phylogeographically structured across their former range? Is the genetic diversity of captive addax populations representative of their historical diversity? The answers to these questions are not only interesting from an evolutionary and historical point of view, but also have major implications for the present and future conservation of the species.

In this study, we provide baseline information about the genetic diversity and phylogeography of historical addax populations by examining complete mitochondrial genomes from across its historical range. We also present, to our knowledge, the first nuclear genome assembly for the species and reconstruct its effective population size during the Pleistocene. In addition, we present an estimate for the nuclear diversity and inbreeding status of an individual from the European zoo population.

## 2. Materials and Methods

### 2.1. Samples

One contemporary sample of an adult female addax that died of natural causes at Tierpark Berlin was obtained from the Institute of Zoo and Wildlife Research, Germany (Table 1). Bone or skin samples from ten historical specimens from four countries and five different locations collected between 1821–1926 were obtained from the mammal collection of the Museum für Naturkunde, Berlin (Table 1, Figure 1).

### 2.2. Laboratory Procedures

#### 2.2.1. Nuclear Genome

**DNA Preparation.** Genomic DNA was extracted from a contemporary liver sample of a female addax following the standard protocol of the Qiagen DNeasy Blood & Tissue Kit. The DNA was then sheared using a Covaris S220 sonicator (peak incident power: 105, duty factor: 5%, cycles per burst: 200, treatment time: 80 s) aiming for a fragment size of 500 bp. These fragments were then built into double-stranded libraries following Meyer and Kircher [31] with modifications from Fortes and Paijmans [32]. To determine the optimal number of amplification cycles for the subsequent dual-indexing PCR, a qPCR (Thermo Scientific PikoReal Real-Time PCR System) was performed. A library size-selection was performed aiming for 500–1000 bp fragments using the Pippin Prep (Sage Science) standard protocol for 2% Agarose Gel Cassettes for targets between 100–600 bp (Marker B). Sequencing was performed in three independent runs on an Illumina NextSeq500 at the University of Potsdam, Germany, producing 75 and 150 bp paired-end reads.

#### 2.2.2. Mitochondrial Genomes

**DNA Preparation.** DNA was extracted from bone samples using the protocol established by Dabney et al. [33] and from skin samples using a combination of the Rohland et al. [34] protocol with a modified digestion buffer following Taron et al. [35] (5 M GuSCN, 25 mM NaCl, 50 mM Tris, 20 mM EDTA, 1% Tween-20, 1% 2-Mercaptoethanol) and Dabney et al. [33]. For each sample, single-stranded libraries following Gansauge and Meyer [36] were prepared from 20 µL of the DNA extract, including a uracil removal step with Uracil-DNA glycosylase (Afu UDG) and Endonuclease VIII, which cuts abasic sites. The optimal number of amplification cycles for dual-indexing PCR was determined using qPCR. For sample ZMB MAM 35370, two libraries from two different samples were built. To control for contamination, extraction and library blanks were processed alongside all samples. All pre-PCR lab work was carried out in dedicated archival DNA facilities at the University of Potsdam, Germany.

**Mitochondrial Genome Enrichment.** An Agilent SureSelect Array was designed using the available mitochondrial genome of *A*. *nasomaculatus* from GenBank (JN632591 [37]) and dividing it into 60-mer probes with 3-bp tiling using a custom python script. For sequence capture, the samples were divided into two batches, which were captured separately with the same array. The libraries were combined for each batch so that the samples were pooled in equimolar amounts (except one sample in batch two), which led to a total input amount of 1649.34 ng of DNA for batch one and 892.63 ng of DNA for batch two (Tables S9 and S10). Two rounds of array capture were performed for each batch [32,38]. The libraries were reamplified after each capture round using the Agilent Herculase II Fusion DNA Polymerase with 24 and 13 cycles for batch one and 23 and 17 cycles for batch two. Cycle numbers were estimated using qPCR to avoid overamplification. Subsequently, the libraries were sequenced in two runs on an Illumina NextSeq500 at the University of Potsdam, producing 75 bp single-end and 75 bp paired-end reads.

### 2.3. Bioinformatic Procedures and Analyses

#### 2.3.1. Nuclear Genome

**Read Preparation.** Paired-end reads from all three sequencing runs were simultaneously merged and adapter sequences were removed with Seqprep v1.1 (https://github.com/jstjohn/SeqPrep) using the parameters -q 13 -L 30. Quality trimming was carried out with Sickle v1.33 (https://github.com/najoshi/sickle) using a quality value of 32. Finally, quality-trimmed sequences were corrected using BFC kmer correction v1 [39] and selecting a kmer size of 51.

**Genome Size Sequencing Coverage Estimation.** Multiplicity distribution of 23-mers was carried out with Jellyfish2 v2.2 [40] and KrATER v0.35 (https://github.com/mahajrod/KrATER) in order to estimate coverage.

**Genome Assembly.** All reads were mapped with BWA using the mem algorithm v0.7.13 [41] to chromosomes of the domestic goat (*Capra hircus,* GCA_000317765.2 [42]) and the Y chromosome of the wild goat (*C*. *aegagrus*, CM003213.1 [43]). A custom set of in silico mate pairs was generated from the consensus genome using cross-mates v1.0 [44]. Genome assembly was then carried out using SOAPdenovo v2.04 [45] with the trimmed error-corrected reads and the in silico mate pairs produced via mapping to the domestic goat assembly using cross-mates. The most continuous assembly was obtained with the following parameters during sparse_pregraph: -K 61 -z 4000000000 -g 15 -d 5 -e 5 -R -r 0; and using the minimal merging strength during contig building. Gaps in scaffolds were filled using GapCloser v1.12 with default parameters [45]. Finally, all scaffolds <1000 bp were removed from the assembly. Quast v5.0.2 [46] and BUSCO v5.1.3 [47,48]) with default settings in offline mode, with the Cetartiodactyla (*N* = 13,335 genes), the Laurasiatheria (*N* = 12,234 genes), and the Mammalia (*N* = 9226 genes) BUSCO lineage datasets (odb10), were used to determine the quality and completeness of the addax assembly, respectively.

**Contamination Check of Assembly.** The draft assembly was split into 5-kb segments with an overlap of 100 bp using GenomeTools shredder v1.5.7 [49] and subsequently BLASTed against the NT database using BLAST [50].

**Pairwise Sequential Markovian Coalescent Model.** For the pairwise sequential Markovian coalescent (PSMC) model, only the raw reads from the two sequencing runs with 150 bp paired-end data were used, while the test run with 75 bp paired-end data was omitted. Illumina adapter sequences were trimmed (overlap 1 bp) from the raw reads using Cutadapt v2.8 [51] and reads shorter than 30 bp were removed. Overlapping reads were merged using FLASH v1.2.11 [52] with a maximum overlap of 150 bp. The resulting merged and unmerged reads were mapped to the genome of the scimitar-horned oryx (*Oryx dammah*) [53] with the BWA mem algorithm v0.7.17 [41]. It was decided to use the genome of the scimitar-horned oryx as opposed to our newly constructed addax assembly because recent research indicates that references built using cross-species scaffolding could influence demographic analyses using the PSMC model [54]. SAMtools view v1.10 [55] was used to filter reads with a mapping quality of <30 and duplicates were removed with Picard MarkDuplicates v2.22.0 (Picard Toolkit 2020—http://broadinstitute.github.io/picard).

Sex chromosomes are likely to differ in their demographic history compared to autosomes [56]. Therefore, scaffolds likely to represent X and Y chromosomes were identified by aligning the scimitar-horned oryx genome to the X chromosome of the domestic goat (*C*. *hircus*, CM001739.2 [42]) and the Y chromosome of the wild goat (*C*. *aegagrus*, CM003213.1 [43]) as well as the complete mitochondrial genome of the scimitar-horned oryx (JN632677 [37]) using SatsumaSynteny v2.0 [57]. 53 scaffolds were identified and removed from downstream analyses (Table S6**)**.

The mutation rate per site per year for the addax and the scimitar-horned oryx were inferred by using the mapped data of both species to the scimitar-horned oryx genome. Subsequently, the genetic distance between the two species was determined using ANGSD v0.923 [58]. Following the estimated split of *Addax* and *Oryx* at 2.21 Mya in the dated mitochondrial species phylogeny (see below), the mutation rate per site per year was inferred to be 1.604 × 10^−9^ between the two species (Table S14). For plotting the population dynamics on an absolute time scale, a generation time of 6.8 years for the addax [59] and 6.2 years for the scimitar-horned oryx [60] were used.

A diploid consensus sequence of the autosomal scaffolds of the scimitar-horned oryx (-R, using a bed file) was created using a combination of bcftools v1.10 mpileup (-Ou, -C0), call (-c) and vcfutils [61,62] (minimum read depth -d 17, maximum read depth -D 103). Then, fq2psmcfa was used to generate the PSMC input file using a quality cutoff of 20 (-q). Finally, the PSMC model v0.6.5 [56] was applied with parameters previously shown to be relevant with human data (-N25 -t15 -r5 -p “4 + 25 × 2 + 4+6”) to infer changes in effective population size (N_e_) through time. With splitfa (from the psmc package), long sequences of the input file were broken down and sampled randomly with replacement to perform bootstrapping analyses using 100 replicates. The results were plotted using a generation time of 6.8 years [59] and a per-generation mutation rate of 1.09 × 10^−8^, based on the yearly per site mutation rate estimated in this study, to scale the x axis. Mutation rates per year for the 95% HPD minimum and maximum interval of the split from the same analysis were also calculated and used to plot the alternative results (Table S14 and Figures S1–S3).

For comparison, a PSMC analysis for the scimitar-horned oryx (*O*. *dammah*) was also conducted, which inhabits a similar environment as the addax. Its raw data was treated in the same way as that of the addax (with the difference of a maximum overlap FLASH parameter of 145 bp) and also mapped to the scimitar-horned oryx genome [53]. For the PSMC, only the vcfutils (minimum read depth -d 23, maximum read depth -D 136) parameters were adjusted. For plotting, a generation time of 6.2 years [60] and a per-generation mutation rate of 9.95 × 10^−9^ were used. PSMC trajectories of both species were plotted together on a logarithmic and a linear time scale using R v3.6.3 (https://www.R-project.org) [63], R Studio v1.4.1106 (https://www.rstudio.com) [64]), and Inkscape v0.91 (https://inkscape.org).

**Nuclear DiversityComparison.** The autosomal heterozygosity of the addax was compared to that of other ungulate species. Six wild ungulate species with IUCN Red List categories ranging from Near Threatened to Extinct in the Wild and with different distributions and body sizes were chosen: scimitar-horned oryx (*O*. *dammah*), gemsbok (*O*. *gazella*), sable antelope (*Hippotragus niger*), African buffalo (*Syncerus caffer*), springbok (*Antidorcas marsupialis*), and Defassa waterbuck (*Kobus ellipsiprymnus*) (Table S3 [53,65,66,67]). All raw reads were treated as described above for the PSMC analysis with the exception of the African buffalo, the springbok, and the Defassa waterbuck, which were already pre-trimmed and therefore did not require adapter trimming with Cutadapt. The maximum overlap parameter during merging of paired-end reads with FLASH was varied according to the longest read length. Each species was mapped to the assembly generated from the respective raw reads. The resulting bam files after duplicate removal were subsampled with SAMtools view v1.10 [55] to an average coverage of 23x to avoid possible biases resulting from uneven coverage. Sex chromosomes and mitochondrial genomes were determined for each nuclear reference with SatsumaSynteny v2.0 [57] using the X chromosome of the domestic goat (*C*. *hircus*, CM001739.2 [42]) and the Y chromosome of the wild goat (*C*. *aegagrus*, CM003213.1 [43]), which both have the same phylogenetic distance to the comparison species, and the mitochondrial genome of the respective species (Table S6 [37]). All identified scaffolds as well as all scaffolds shorter than 1 Mb were excluded from the analysis to avoid misalignments of short scaffolds. To estimate the autosomal heterozygosity for each species, allele frequencies were calculated using genotype likelihoods in ANGSD v0.923 [58] following Westbury et al. [68], setting the -setMaxDepth parameter to twice the average coverage (45) but using -C 50 instead of 0 (with parameters per species -minInd 1, -setMinDepthInd 5, -doCounts 1, -GL 1, -doSaf 1, -fold 1, -minQ 30, -uniqueOnly 1, -remove_bads 1, -only_proper_pairs 1, -baq 1 [69]). Subsequently, realSFS in ANGSD v0.923 was used to calculate the site frequency spectrum. Standard deviations (SDs) were estimated for window sizes of 20-, 50-, and 100-Mb sites across the whole genome using the -nSites option in ANGSD v0.923 (Table S4). In addition, the heterozygosity for non-overlapping 500-kb sliding windows for the autosomal scaffolds was calculated to estimate its distribution for each species using the -r option in realSFS (-tole 1 × 10^−8^) [70]. Only windows with less than 60% missing data were considered and values <0.001 were binned as 0. The results were plotted using R v3.6.3 (https://www.R-project.org) [63], R Studio v1.4.1106 (https://www.rstudio.com) [64], and Inkscape v0.91 (https://inkscape.org).

**Inbreeding Assessment.** ROHan [71] was run using the bam files generated from mapping reads from our addax individual to our newly constructed addax genome assembly (see above) with default parameters (window size 1 Mb, expected theta in ROHs <1 × 10^−5^) to determine genome-wide heterozygosity and runs of homozygosity (ROH), which provide a measure for recent inbreeding [72]. Only autosomal scaffolds larger than 1 Mb (--auto) were included in the analysis. The input bam file was filtered with SAMtools view v1.10 [55] for unmapped reads and reads failing the vendor quality check (-F516). Duplicates were removed using Picard MarkDuplicates v2.22.0 (Picard Toolkit 2020—http://broadinstitute.github.io/picard). The analysis was also run for the scimitar-horned oryx, mapping reads to its available genome assembly [53] and filtering the bam file in the same way as was done for the addax.

#### 2.3.2. Mitochondrial Genomes

**Read Mapping.** Data from different sequencing runs were combined for each specimen before processing (ZMB MAM 35370, IZW 607/10) (Supplementary File S1: Text S2). Illumina adapter sequences were trimmed (overlap 1 bp) and reads shorter than 30 bp were removed using Cutadapt v2.8 [51]. For the paired-end data, overlapping reads were merged using FLASH v1.2.11 [52] with a maximum overlap of 75 bp for historical and 150 bp for contemporary data. Unmerged reads were discarded. The trimmed and merged reads were mapped to the available addax mitochondrial genome (JN632591, from an animal at the Parc Zoologique de Paris, France [37]) using BWA v0.7.17 with the aln algorithm [41] and default settings. Reads with a mapping quality of <30 were filtered out with SAMtools view v1.10 [55] and duplicates removed with MarkDupsByStartEnd v0.2.1 (https://github.com/dariober/Java-cafe/tree/master/MarkDupsByStartEnd). All historical specimens showed a tandem repeat of ACAT in the control region in comparison to the reference (JN632591) and the contemporary specimen (IZW 607/10). Therefore, a manual editing step of the bam files of all historical specimens was conducted to correct for the lack of this tandem repeat in the reference genome which resulted in the correct ACATACAT section in the final consensus sequence. A consensus sequence was generated using an 85% majority rule threshold for base calling, a minimum coverage of 3× and the “trim to reference” option using Geneious R10 v10.2.3 [73] (https://www.geneious.com). To enhance coverage at the edges of the sequences, the reads were mapped and filtered again in the same way as stated above to a reference in which the last 400 bp were shifted from the end to the beginning of the reference, making use of the circular character of the mitochondrial genome. For all samples except IZW 607/10, the bam files were again edited manually, and consensus sequences called. For all consensus sequences, the part corresponding to the shifted 400 bp was moved back to the end of the sequence. Both consensus sequences were then combined using a 50% majority rule threshold for base calling (option “50%—Strict: Bases matching at least 50% of the sequences”). This resulted in enhanced coverage at the edges for all individuals except ZMB MAM 2166, ZMB MAM 35370, and IZW 607/10.

**Phylogenetic Analysis.** Complete mitochondrial genome sequences for 13 wild ungulate species were compiled from Genbank and the control region removed for alignability: scimitar-horned oryx (*O*. *dammah*), Arabian oryx (*O*. *leucoryx*), East African oryx (*O*. *beisa*), Gemsbok (*O*. *gazella*), blue antelope (*H*. *leucophaeus*), sable antelope (*H*. *niger*), roan antelope (*H*. *equinus*), topi (*Damaliscus lunatus*), blesbok (*D*. *pygargus phillipsi*), hartebeest (*Alcelaphus buselaphus*), black wildebeest (*Connochaetes gnou*), blue wildebeest (*C*. *taurinus*), and greater kudu (*Tragelaphus strepsiceros*) (Table S13 [37,74,75,76]) (alignment length: 15,657 bp, Supplementary File S3). To date the basal divergence of the addax mitochondrial haplotype lineage, the two most diverged addax sequences (Figure 6) were included into this alignment. Hereafter this alignment is called the species alignment to distinguish it from the alignment with only addax sequences, which is called the population alignment. For the population alignment, the control region was retained. Both alignments were saved in NEXUS format for analysis in BEAST v2.5.0 [77,78] with ambiguities indicated in IUPAC code. Alignments of the population and species datasets were carried out in Geneious R10 v10.2.3 using the MAFFT algorithm v7.450 [79,80] with default settings.

**Phylogenetic Network.** From the population alignment, all ambiguities/missing data and gaps were removed, resulting in an alignment length of 16,685 bp (Supplementary File S5). A phylogenetic network was reconstructed with POPART v1.7 [81] using the TCS algorithm [82,83] and edited in Inkscape v0.91 (https://inkscape.org). In addition, the number of segregating sites and nucleotide diversity were determined.

**Mitochondrial Diversity Comparison.** The mitochondrial diversity of addax was compared with that of seven wild ungulate species using a pairwise diversity comparison. Seven mitochondrial genomes (including control region) per species were randomly chosen from all contemporary or historical non-hybrid sequences from GenBank to represent each species (Table S15 [37,53,67,84,85,86,87,88,89,90]). For scimitar-horned oryx, one sequence and for European bison, two sequences were excluded that were already deemed problematic by other studies [89,91]. For each species, sequences were aligned using the MAFFT algorithm v7.450 [79,80] with default settings in Geneious R10 v10.2.3 [73] (https://www.geneious.com). Next, overall average pairwise distances (k) were calculated in MEGA X [92] with gaps and missing data treated as complete deletions. For this comparison, mitochondrial genomes of *H*. *niger* were assembled from the available raw whole genome data [67] (Supplementary File S1: Text S4, MZ488448-MZ488453). Results were plotted using R v3.6.3 (https://www.R-project.org) [63], R Studio v1.1.423 (https://www.rstudio.com) [93], and Inkscape v0.91 (https://inkscape.org).

**Bayesian Mitochondrial Species Phylogeny.** Analysis parameters and priors were specified in BEAUTi v2.5.0. The analysis was carried out in BEAST v2.5.0 [77,78,94]. This analysis included all extant hippotragine and alcelaphine species, as well as the greater kudu (*T*. *strepsiceros*) in a species alignment (see above, alignment length: 15,657 bp, Supplementary File S3). JModeltest v2.1.10 [95,96] was run with five substitution schemes, allowing models of equal/unequal base frequencies, with and without a proportion of invariable sites, with and without rate variation among sites, allowing six gamma categories, and using "BIONJ" as the base tree for likelihood calculations. Using the corrected Akaike Information Criterion (AICc), the GTR+I model was found as the best-fitting substitution model. However, the GTR+G (second best-fitting model) was chosen because the use of gamma categories already permits sites with very low substitution rates [97]. Furthermore, no partitioning of the mitochondrial genomes was conducted because the specified six gamma categories permit fast evolving and slow evolving regions to fall into higher and lower categories, respectively [98]. Site frequencies were inferred empirically from the dataset and ambiguities were accounted for (option "use ambiguities" in BEAUTi). A relaxed lognormal clock was applied to allow for substitution rate variation between different lineages. A Yule model was chosen as the tree model prior. A uniform prior between 0 and 1 was set on the clock rate. Five node calibrations were used to calibrate the tree following mostly Bibi [27] with one improvement for the crown *Connochaetes* spp. node (Table 2, blue circles in Figure 4) (see Supplementary File S1: Text S3: Fossil Calibrations [27,37,75,99,100,101,102,103,104,105,106]). All calibration priors were set to be monophyletic based on previous phylogenetic analyses [27,37]. To determine the split age between *Oryx* and *Addax* and to date the basal divergence of the mitochondrial haplotype lineage of the addax individuals sampled, an uninformative prior was set on each of these two nodes (marked with stars in Figure 4). The parameter popSize of the starting tree had to be manually adjusted in the xml file from 1.0 to 1000.0, to be able to start the analysis. The MCMC was run for 60 million generations. Trees were sampled every 6000 generations and were summarized using maximum clade credibility as the target tree type and median node heights in TreeAnnotator v2.5.0 [77,78] after a burn-in of 10%, which was chosen following visual inspection of the results in Tracer v1.7.1 [107]. The tree was visualized using FigTree v1.4.3 (https://github.com/rambaut/figtree) and Inkscape v0.91 (https://inkscape.org).

**Bayesian Addax Mitochondrial Phylogeny.** This analysis was restricted to only include the addax individuals sampled in this study and one sequence from GenBank. Twelve complete addax mitochondrial genomes, including the control region (alignment length: 16,758 bp, Supplementary File S4) were included. jModeltest v2.1.10 [95,96] using AICc found that HKY+I+G was the best-fitting substitution model. For the reasons mentioned above, only HKY+G was chosen, no partitioning of the mitochondrial genome was executed, six gamma categories were allowed for, site frequencies were empirically estimated from the dataset, and ambiguities in the dataset were considered (option “use ambiguities” in BEAUTi). Since only addax sequences were included, a strict clock was chosen. An exponential coalescent population model prior was used to account for possible population growth or decline. Following the age estimate for the addax individuals analyzed in the species phylogeny, the root age was calibrated with a lognormal prior using the inferred age (mean 32 ka, 95% credibility interval (CI) 11–58 ka) for the most recent common ancestor (MRCA) of the two addax individuals used in the mitochondrial species phylogeny. The MCMC was run for 50 million generations and trees sampled every 5000 generations. The maximum clade credibility tree with median posterior node heights was generated using TreeAnnotator v2.5.0, with a burn-in of 10% after visual inspection of the log file in Tracer v1.7.1. The tree was visualized using FigTree v1.4.3 (https://github.com/rambaut/figtree) and Inkscape v0.91 (https://inkscape.org).

## 3. Results

### 3.1. Assembly of Nuclear Genome and Mitochondrial Genome Data

Using shotgun sequencing and hybridization capture approaches, we obtained a 44× coverage nuclear genome of a contemporary addax zoo individual, ten mitochondrial genomes from museum specimens, and one mitochondrial genome from the same zoo individual, ranging from 62.58× to 7907.87× coverage. Table 3 gives basic assembly statistics for the addax nuclear assembly. BUSCO analysis ranked the addax assembly with a high degree of completeness for the Cetartiodactyla (91.6%), the Laurasiatheria (92.4%), and the Mammalia gene sets (91.2%) (Table 4).

More specific details on read numbers can be found in Tables S1, S2, S11, and S12 and on the assembly in Supplementary File S1: Text S1.

#### 3.1.1. Genetic Diversity and Demographic History

**Figure 2:**
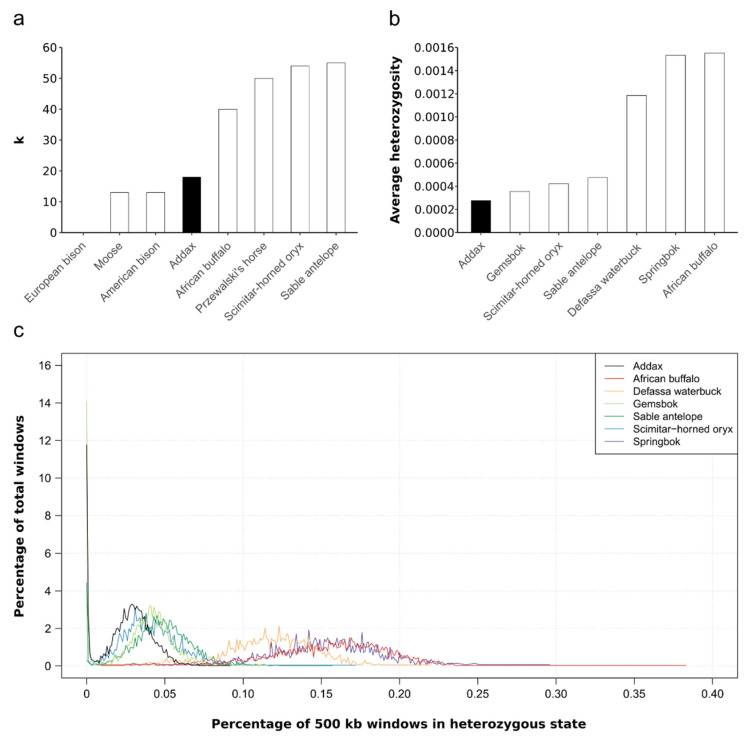
**Comparisons of mitochondrial and nuclear diversity**. (**a**) Comparison of the overall average pairwise distance of the addax (*Addax nasomaculatus*) with seven wild ungulate species using seven complete mitochondrial genome sequences per species. k gives the average number of substitutions between two individuals of the same species. Note that the value for the European bison is zero. (**b**) Comparison of the average autosomal heterozygosity of the addax with six wild ungulate species. (**c**) Heterozygosity for 500-kb sliding windows across the autosomal scaffolds of seven wild ungulates species. The addax is marked in black in all panels.

Comparison of the addax to seven other wild ungulate species (Figure 2a) showed that the addax had a relatively low mitochondrial diversity, similar to that of American bison or moose, but much lower than its close relatives the scimitar-horned oryx and the sable antelope (Table S16). Moreover, comparison of the average autosomal heterozygosity of the addax and six wild ungulate species revealed a relatively low level of nuclear diversity for the addax (Figure 2b). Heterozygosity estimates from 500-kb sliding windows across the genome showed a similar picture (Figure 2c): the addax, together with the three other hippotragine species, displayed a more limited heterozygosity distribution than the Defassa waterbuck, springbok, and African buffalo. Both the addax (11.8%) and the gemsbok (14.1%) had a higher percentage of total windows with very low levels of heterozygosity (<0.001) than the other species. Standard deviations are displayed in Table S5.

#### 3.1.2. Inbreeding Assessment

Based on the default definition that a ROH is a region of at least 1 Mb with an average heterozygosity <1 × 10^−5^, no runs of homozygosity (0%) were found within the addax genome assembly (Table S7) using ROHan. However, for the scimitar-horned oryx, 9.05% of the genome was in ROH with the average length of a ROH being 4,666,670 bp (Table S8).

#### 3.1.3. Pairwise Sequential Markovian Coalescent Model

The autosomal pairwise distance between the scimitar-horned oryx and the addax was estimated to be 0.007091. Based on the 2.21 Mya divergence age estimate between *Oryx* and *Addax,* a mutation rate of 1.604 × 10^−9^ per year and a mutation rate of 1.09 × 10^−8^ per generation, assuming a generation time of 6.8 years [59], was calculated. This is almost identical to the mutation rate of 1.1 × 10^−8^ used in Humble et al. [53] for the scimitar-horned oryx.

**Figure 3:**
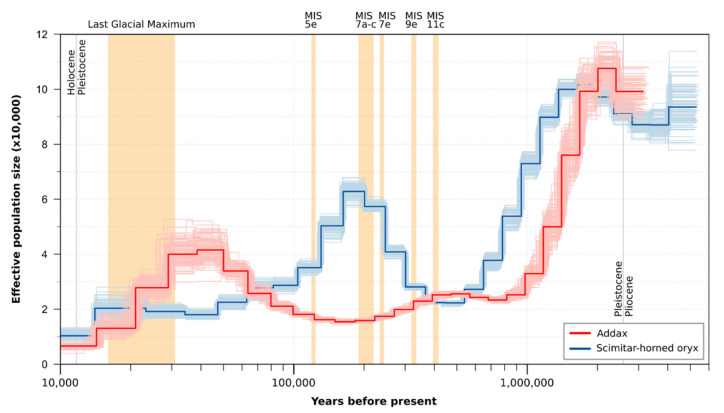
**Pairwise sequential Markovian coalescent model for the addax and the scimitar-horned oryx.** Changes in effective population size N_e_ (with 100 bootstrap repetitions) in the addax and the scimitar-horned oryx based on an autosomal pairwise sequential Markovian coalescent model (addax: generation time 6.8 years, mutation rate per generation (µ) 1.09 × 10^−8^ [59], this study; scimitar-horned oryx: generation time 6.2 years, mutation rate per generation (µ) 9.95 × 10^−9^ [60], this study). Last Glacial Maximum and Marine Isotope Stages (MIS) representing interglacial periods of the last 500,000 years are marked in light orange. Note the Late Pleistocene reduction in population size, approximately coinciding with the age of the most recent common ancestor of the mitochondrial genome samples (Figure 4 and Figure 5). Scimitar-horned oryx data from Humble et al. [53]. See Figure S1 for a presentation of the results on a linear time scale.

**Figure 4:**
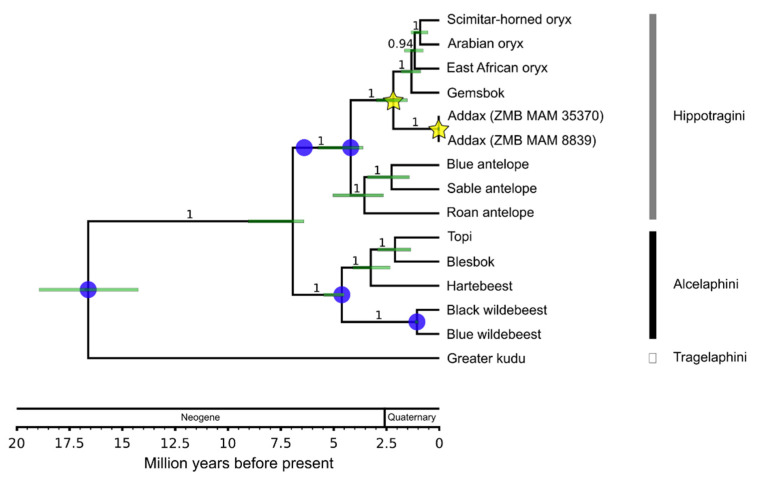
**Calibrated mitochondrial species phylogeny.** Branch values show Bayesian posterior probabilities, light green node bars represent 95% credibility intervals of divergence time. Blue circles mark the five fossil calibrations used. Yellow stars mark the *Addax*/*Oryx* divergence (1.51–2.98 Mya) and the most recent common ancestor of the addax samples (11–58 kya, 95% credibility interval).

The PSMC trajectory of the addax is characterized by an overall continuous decline of effective population size (N_e_) over time with the exception of a short but steep increase beginning ~150 kya and peaking ~44 kya before steeply declining through the Last Glacial Maximum to the present (Figure 3). When compared with the trajectory of the scimitar-horned oryx, the N_e_ of the two species developed inversely during two time periods. It declined for both species up to ~620 and ~490 kya, respectively. After this, N_e_ of the addax increased very briefly and then decreased, whereas the N_e_ for the scimitar-horned oryx increased considerably until it started to decline again ~150 kya, when the N_e_ of the addax increased again.

### 3.2. Divergence Age and Phylogeographic Structure

#### 3.2.1. Species Phylogeny

**Figure 5:**
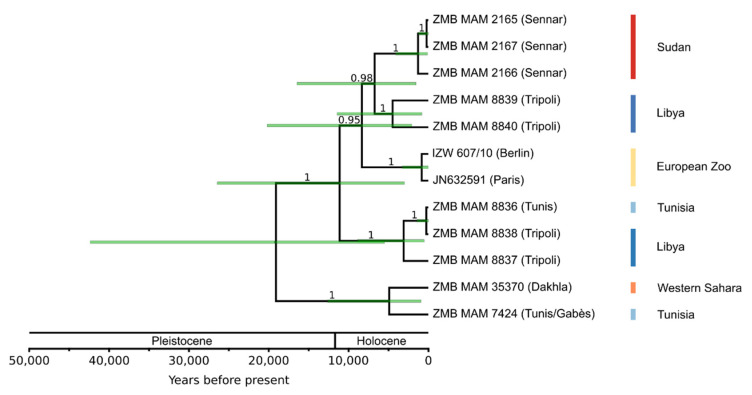
**Secondarily calibrated Bayesian phylogeny of twelve addax mitochondrial genomes.** The root age was calibrated using the age estimate from the Bayesian mitochondrial species phylogeny (Figure 4). Branch values show posterior probabilities, light green node bars represent 95% credibility intervals of divergence times. The lower genetic variability among addax specimens resulted in large uncertainty of node ages.

The calibrated Bayesian species phylogeny dated the divergence of *Addax* and *Oryx* to 2.21 Mya (1.51–2.98 Mya, 95% CI) and the age of the MRCA of the two most divergent addax haplotypes to 32 kya (11–58 kya, 95% CI) (Figure 5). Effective sample size (ESS) values for the tree likelihood, priors, and input parameters were >700 for the species analysis.

#### 3.2.2. Population Phylogeny

The addax population phylogeny (Figure 5) showed a monophyletic Sudan clade, but highly intermingled relationships among haplotypes from the Tunisian, Libyan, and Western Saharan individuals. All ESS values were >1000 for the population analysis.

**Figure 6:**
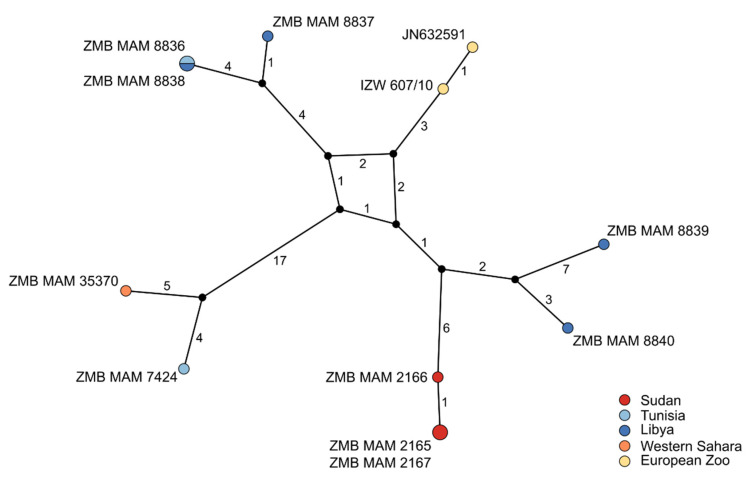
**TCS network of twelve addax mitochondrial genomes.** The TCS network of the complete mitochondrial genomes generated in POPART v1.7 takes only positions into account that are present in all sequences while excluding ambiguities/missing data (alignment length: 16,685 bp). Numbers and black circles represent substitutions and unsampled haplotypes, respectively. Circle size corresponds to the number of specimens assigned to a haplotype.

The TCS phylogenetic network of the twelve complete addax mitochondrial genomes showed 59 segregating sites and a nucleotide diversity of 0.0007 (Figure 6). While some geographic structure is present, this is evidently weak, as shown by the lack of grouping of samples from Tunisia and Libya. The close maternal relatedness of the two zoo individuals (IZW607/10 and JN632591), and the identical sequences of two individuals from Sudan (ZMB MAM 2165 and 2167) and two from Tunisia and Libya (ZMB MAM 8836 and 8838) were also evident.

## 4. Discussion

Genetic investigations of species on the verge of extinction can be of vital importance. Here we showed that genetic and genomic methods coupled with analyses of museum samples using archival DNA methodologies can reveal baseline information such as population demography and past and present diversity. In the case of the addax, we show that this species likely had a historically higher mitochondrial diversity than today and relatively weak population structure (Figure 5 and Figure 6). We estimated an age of divergence from *Oryx* spp. at ~2.21 Mya, which is younger than previous estimates (e.g., Bibi [27]), and we inferred an almost continuous decline in effective population size since ~2 Mya (Figure 3). Despite a small population size in recent times, we detected no signs of inbreeding for the zoo individual.

### 4.1. The Addax Exibits Low Nuclear and Intermediate Mitochondrial Diversity

Hippotragini seem to have a lower nuclear diversity than other African bovids, with the addax having the lowest nuclear diversity among them in our comparison. While some of these African bovids have smaller geographic ranges than the addax, they almost certainly have had consistently higher population sizes on account of their more productive habitats. Having relatively low nuclear diversity might be a characteristic of Hippotragini independent of whether they inhabit a desert environment or not. However, all four hippotragin genomes analyzed in our study originated from zoo animals. Since quality of pedigrees and management of the captive populations might differ, the genome-wide diversity of these animals might not be representative of wild (or formerly wild in the case of the scimitar-horned oryx) populations.

Our mitochondrial DNA results indicate that the 19th and 20th century population of wild addax showed intermediate levels of mitochondrial diversity compared to several other wild ungulates and low levels compared to African bovids in general. Although our sample size only included ten wild individuals, it does represent the peripheries of the former geographic range of this species, which once covered the entire Sahelo–Saharan region, an area of ~9.5 million km^2^ (about the size of Brazil or China, Figure 1). Species with lower mitochondrial diversity than the addax include the European and American bison, which are both known to have experienced recent bottlenecks [116,117] (~140 years ago [118]) but also the moose, which did not go through a significant reduction in population size. However, species with a much higher mitochondrial diversity include on the one hand the scimitar-horned oryx and Przewalski’s horse, which have both recently been severely reduced in population size [119,120], but on the other hand, the African buffalo and the sable antelope, which like the moose, have not suffered such a reduction. This might point to a limited connection between mitochondrial diversity and population size, as postulated by Bazin et al. [121]. However, the addax appears to have also gone through a recent period of relatively low population size, as our dated species phylogeny placed the age of the MRCA at ~32 ka (11–58 kya, 95% CI). The credibility interval on this age estimate is relatively large, but we can be certain that major losses in addax mitochondrial diversity occurred well before the oldest specimens in our sample (1821), and well before modern human pressures on the Sahelo–Saharan region, including hunting with firearms and poaching. The MRCA age estimate also corresponds to a major decline in effective population size in the PSMC trajectory shortly after ~44 ka, which suggests that a major decrease in the addax effective population size took place in the Late Pleistocene, probably between ~50 kya and the Last Glacial Maximum. Therefore, the addax joins the growing list of large mammals for which genetic evidence indicates major population size decreases during the last 100 kya [65,122]. In summary, the addax appears to have had a small population size in both historical and prehistoric times, prior to the human disturbances of the last few centuries. A major decrease in numbers occurred during the Late Pleistocene as part of a global wave of large mammal extinctions and extirpations, for which global (rather than local) explanations might be sought.

### 4.2. Weak Phylogeographic Structure and Climatic Influence on Population History

Previous work has shown the existence of measurable phylogeographic structure among sub-Saharan African large mammals [123]. Lerp et al.’s [124] analysis of Saharan and Arabian dorcas gazelles (*Gazella dorcas*) found low genetic diversity and no clear phylogeographic structure across its range. A study on dama gazelles (*Nanger dama*) also found only weak phylogeographic structure [125]. Similar to those two desert species, we found that phylogeographic structure in the addax is low.

The addax mitochondrial genomes are distinguished by only a few differences (Figure 6). The highest diversity can be seen between North and West African individuals, and it is notable that North African individuals are paraphyletic (Figure 5). The European zoo clade is reported to descend from a founding population of 15 individuals [59]. The mother and grandmother of our zoo individual were born in Tierpark Berlin. The father and grandfather are from the San Diego Zoo. Animals from Berlin can be traced back to two founders from Chad and two other individuals of unknown origin [126]. The Chad origin of the maternal relatives of the Berlin individual matches with the results marking the origin of its mitochondrial lineage to somewhere between Libya and Sudan (Figure 5 and Figure 6).

The absence of deep phylogeographic structure observed in the addax suggests a lack of barriers to dispersal and gene flow across its former range and that addax may have originally consisted of a single gene pool, consistent with its high mobility and nomadic behaviour [127]. This makes sense considering the large foraging areas that must have been required to sustain herds in the hyperarid environments and meagre vegetational resources of the Sahara. Former reports indicating that the addax would temporarily aggregate in groups of “several hundreds” of individuals, although usually living “in small herds of up to 15 animals” [127], show how successful they were at this.

As the PSMC method rapidly loses resolution as it moves toward the present, any correspondence with multiple climatic cycles with a periodicity of ~100 kyr or less is difficult to test. However, we did see some fluctuations over the last 500 kyr, with peaks in effective population size at ~500 and ~44 kya. We can only speculatively attempt to relate these to climatic changes, in part because the climatic history of the Sahara remains poorly resolved. For example, while previous work had proposed arid–dry cycles in this region at 100 ky periodicity corresponding to that of Northern Hemisphere glacial cycles (e.g., de Menocal [128], Dupont [129]), a more recent assessment of the Saharan dust record suggests dominance of 23 ky (precessional) climatic cycles [130]. Moreover, while previous work on lake core records had proposed a pan-African period of “megadrought” between 135 and 70 ka [131,132], Saharan records in fact indicate the presence of humid habitats at 120 ka, and probably at several times during previous interglacials [133,134,135]. Within this context, it could be that addax population size decreased (or that gene flow across its range was disrupted) with the advent of humid conditions during the last interglacial (~120 ka) and increased again during the last glacial period (Figure 3). The fact that this pattern appears to be inverted in the scimitar-horned oryx may also support this interpretation. In contrast with the truly Saharan addax, the scimitar-horned oryx historically resided in the semi-arid grasslands along the peripheries of the Sahara in the Sahel and North Africa [14,136]. Our PSMC results suggest that the scimitar-horned oryx might have benefited from Pleistocene humid periods, during which its grassland range might have expanded deep into the Sahara. In contrast, it would appear that the addax fared poorly during these times of increased productivity, which would be surprising. Perhaps the addax is so specialized to its desert habitats that it cannot take advantage of the increased availability of grasses and shrubs, or possibly was outcompeted by encroaching large savanna herbivores. Alternatively, the increased food availability accompanying humid conditions may have decreased the need for addax populations to migrate long distances, thereby potentially restricting gene flow across the Sahara during these times, thereby resulting in a drop in effective population size as measured here.

### 4.3. Conservation Implications

Sahelo-Saharan large mammals such as the addax and the dama gazelle have lost over 90% of their former ranges, and both are on the verge of becoming extinct in the wild [6]. These species potentially face the same fate as the scimitar-horned oryx (*O*. *dammah*), which has been extinct in the wild since 2000 [119]. However, a reintroduction program for this species in Chad that started in 2016 is ongoing and this population is slowly growing in numbers [137]. Many additional Sahelo–Saharan mammal species are similarly threatened, such as the slender-horned gazelle (*G*. *leptoceros*) and barbary sheep (*Ammotragus lervia*) [138,139], escalating the potential extinction crisis facing Sahelo–Saharan wildlife [16,140]. The baseline information about the addax generated in our study can be used to draw several important conservation implications for the species.

First, although we detected ~12% of the 500-kb windows with no heterozygosity in the addax genome (Figure 2c) when using ANGSD, which could indicate inbreeding, we did not detect any inbreeding by means of runs of homozygosity within the nuclear genome of the addax using ROHan. These seemingly contradictory results could be caused by the parameter “-baq 1” (adjusting quality scores around insertions/deletions with normal BAQ calculation) in ANGSD [58], which can lead to a reduction of the overall heterozygosity [54]. Moreover, for the 500-kb window analysis, windows with <0.001 heterozygous sites were binned to 0, which is stricter than in the ROHan analysis (0.00001). Therefore, as ROHan was specifically developed to uncover ROH, and as we also detected ROH in the scimitar-horned oryx showing the ability to detect inbreeding via ROH, we deem the result of no signs of inbreeding in our addax individual to be reliable. This implies that the addax’s relatively low diversity was not caused by recent inbreeding, similar to the cases of brown (*Parahyaena brunnea)* and striped hyenas (*Hyaena hyaena*) [70]. This is a somewhat surprising result since more ROH are expected in small populations [72] and since the addax also shows low overall heterozygosity. However, this is consistent with a study by Brüniche-Olsen et al. [141], which found that IUCN Red List status was not a significant indicator of inbreeding levels. Nevertheless, if the zoo individual we sequenced is representative of the European zoo population as a whole, this would be very good news for addax reintroduction programs that use animals from the European zoo population (e.g., Riordan et al. [142]) and could be seen as a reassurance that the mainly pedigree-based breeding program [143] is able to maintain genomic diversity even with a limited population size. Our zoo individual is a descendant of the European and the American zoo population. Therefore, we would assume that it might be considered as a representative of the zoo population as a whole. However, without insight into the detailed pedigree this question cannot currently be resolved with complete certainty.

Second, the panmictic nature of the historical addax population indicates this species is highly mobile, and confirms that its population ranged widely across the Sahara [2]. Reintroduction programs in small protected areas may therefore prove problematic and very large ranges may be needed to ensure the long-term survival of the addax in the wild (see also Dolan [2]).

Third, only very small populations are left in the wild [1], which are naturally prone to losing diversity through drift. This, together with the lower genomic and possibly low contemporary mitochondrial diversity in the captive population, could indicate that larger amounts of the addax’s diversity might already be lost. However, this is hard to predict from only one investigated nuclear and two contemporary mitochondrial genomes. In addition, nuclear diversity does not decline as fast as mitochondrial diversity, hence only the loss of mitochondrial diversity might be an issue for the addax. An unpublished study of the last wild addax population in Niger suggests that its mitochondrial diversity is higher than that of the captive population [9,142]. Similarly, a study on dama gazelles using mitochondrial markers discovered that this species harbors more diversity in the wild than in captivity [125].

It is important that the diversity of the remaining wild populations is conserved as rapidly as possible, with the goal of establishing a larger wild population comprised of wild and reintroduced individuals. In this case, the conservation dilemma that Senn et al. [125] discuss for the dama gazelle would not apply, since likely only one viable addax population is left in the wild. One way to achieve this could be to locate and capture all remaining wild individuals and include them into local breeding programs together with individuals from zoos and private ranches. Since addax breed well in captivity, this could be a viable way to preserve its remaining diversity if a systematic breeding program is applied. Another option could be to reintroduce animals into the areas where the last wild individuals remain or to other parts of its former range in order to create diverse and demographically stable wild populations. However, both approaches require elimination of the current threats to the addax’s survival in the wild (see Brito et al. [16] for a detailed discussion on how this could be managed), primarily poaching by military personnel accompanying oil and mining companies [14], and habitat degradation due to oil exploration and exploitation [17,20].

The mentioned measures are by far not easy tasks to undertake and have in similar ways already been proposed by the IUCN for the Réserve Naturelle Nationale du Termit et du Tin Toumma in Niger [15]. Certainly, a combined effort including business and governmental authorities as well as conservationists is the most promising way forward [15].

## 5. Conclusions

Through the generation of ten mitochondrial genomes from across the addax’s historical range, and, to our knowledge, the first nuclear genome assembly, our study presented baseline information for a highly threatened desert species. The addax seems to have already had low population sizes in historical and prehistoric times before more recent human interference, which nevertheless is thought to be the reason for its current crisis of severe population decline in the wild. We found only limited evidence for phylogeographic structuring in the historical addax population, suggesting past gene flow and a formerly high degree of mobility across its range. Concerning Pleistocene arid–humid climatic cycles, our data does not give conclusive results but suggests surprisingly that the addax may not have responded positively to the Saharan humid period of the last interglacial. There are indications to suggest that part of the addax’s mitochondrial diversity is already lost, with only very few individuals left in the wild and the zoo population possibly not capturing the historical mitochondrial diversity. However, no or very low levels of inbreeding were detected in the European zoo population, which bears positive implications for reintroduction programs. As conservation measures, we suggest two alternative approaches to preserve as much diversity of the addax as possible. Without rapid action, a species adapted to one of the harshest environments on earth is likely to soon disappear from its natural habitat. Concerted efforts are necessary if we are to ensure the persistence of this species in the wild.

## Figures and Tables

**Table 1:** Historical and contemporary samples of addax (*Addax nasomaculatus*) from the mammal collection of the Museum für Naturkunde, Berlin (ZMB) and the Institute of Zoo and Wildlife Research (IZW), Berlin, Germany. Accession numbers for the mitochondrial genomes generated in this study are listed.

Sample	Country	Location	Collector (s)	Collection Date	Sample Type	Accession Number Mitochondrial Genome
ZMB MAM 2165	Sudan	Sennar	Hemprich and Ehrenberg	1821	bone & skin	MZ474955
ZMB MAM 2166	Sudan	Sennar	Hemprich and Ehrenberg	1821	skin	MZ474956
ZMB MAM 2167	Sudan	Sennar	Hemprich and Ehrenberg	1821	skin	MZ474957
ZMB MAM 35370	Western Sahara	Dakhla	Spatz	1926	bone	MZ474958
ZMB MAM 7424	Tunisia	Tunis, Gabès	Spatz	1884–1903(?)	bone	MZ474959
ZMB MAM 8836	Tunisia	Tunis	Spatz	1884–1903(?)	bone	MZ474960
ZMB MAM 8837	Libya	Tripoli	Browski	1895–?	bone	MZ474961
ZMB MAM 8838	Libya	Tripoli	Browski	1895–?	bone	MZ474962
ZMB MAM 8839	Libya	Tripoli	Browski	1895–?	bone	MZ474963
ZMB MAM 8840	Libya	Tripoli	Browski	1895–?	bone	MZ474964
IZW 607/10	Germany	Tierpark Berlin	-	-	liver	MZ474965

**Table 2:** Fossil calibration priors used with BEAUti/BEAST v2.5.0. Age and 95% ranges were determined as 1.25× the minimum age (lognormal prior) or ± 25% of an approximate age (normal prior). The crown bovid node is calibrated using a normal prior as *E*. *noyei* is presumed to be closely related to crown bovids but its exact relationships to the crown clade remain unclear.

Calibration Point	Prior Type	Age [Mya]	95% Range [Mya]	Fossil Taxon	Site/Geological Unit	References
Crown Bovidae	Normal	18	16.0–20.0	*Eotragus noyei*	Kamlial and Vihowa Formations, Pakistan	[108]
Stem Hippotragini	Log normal	6.4	6.4–8	*Saheloryx tchadensis*, *Saheloryx solidus*, *Tchadotragus sudrei*	Anthracotheriid unit at Toros-Menalla, Chad	[109,110]
Crown Alcelaphini	Log normal	4.5	4.5–5.625	*Damalacra neanica*	Pelletal Phosphorite Member at Langebaanweg, South Africa	[103,111,112]
Crown Hippotragini	Log normal	3.6	3.6–4.5	*Hippotragus* sp., *Oryx* sp.	Lower Laetoli Beds at Laetoli, Tanzania	[113,114,115]
Crown *Connochaetes* spp.	Log normal	1.0	1.0–1.25	*Connochaetes gnou*	Cornelia-Uitzoek, South Africa	[102,105,106]

**Table 3:** Scaffold statistics of the addax in silico mate pair assembly with QUAST v5.0.2.

**Total Assembly Length**	2,795,176,578 bp
**Number of Scaffolds**	86,926
**Scaffold N50**	20,757,513
**Scaffold L50**	37
**Longest Scaffold**	87,765,150 bp
**GC Content**	41.72%

**Table 4:** BUSCO v5.1.3 scores for the addax in silico mate pair assembly using three different BUSCO lineage datasets.

	Cetartiodactyla BUSCO Scores	Laurasiatheria BUSCO Scores	Mammalia BUSCO Scores
**C**	91.6%	92.4%	91.2%
**Complete BUSCOs**	12,209	11,312	8418
**Complete and Single-Copy BUSCOs**	12,038	11,151	8204
**Complete and Duplicated BUSCOs**	171	161	114
**Fragmented BUSCOs**	341	278	287
**Missing BUSCOs**	785	644	521
**Total BUSCO Groups Searched**	13,335	12,234	9226

## Data Availability

The Bioproject number of this project in GenBank is PRJNA742532. The complete addax mitochondrial genomes are available at GenBank with the accession numbers MZ474955–MZ474965 and for the sable antelope with MZ488448–MZ488453 (Sable antelope Bioproject PRJNA403773 and PRJNA403774). The assembly of the addax nuclear genome is available at GenBank under JAIEZW000000000. The untrimmed raw data were uploaded for the addax mitochondrial capture data to the short-read archive under SRR15177977–SRR15177987 and for the nuclear data under SRR15177988, SRR15193276 and SRR15193277.

## Supplementary Materials

The following are available online at https://www.mdpi.com/article/10.3390/genes12081236/s1. Supplementary File S1: Table S1: Paired-end sequencing yields from our contemporary addax sample (IZW 607/10) mapped to the addax nuclear assembly (this study). Table S2: Paired-end sequencing yields from our contemporary addax sample (IZW 607/10) mapped to the scimitar-horned oryx nuclear assembly. Text S1: Assembly Statistics. Figure S1: Pairwise sequential Markovian coalescent model for the addax and the scimitar-horned oryx on a linear scale. Figure S2: Pairwise sequential Markovian coalescent model for the addax with a mutation rate per generation of 1.59 × 10^−8^. Figure S3: Pairwise sequential Markovian coalescent model for the addax with a mutation rate per generation of 8.08 × 10^−9^. Table S3: Accession numbers of raw reads and genome assemblies used in the heterozygosity estimate. Table S4: Estimated autosomal heterozygosity for all scaffolds larger 1 Mb and 20–100-Mb windows in ANGSD v0.923 for the addax and six other wild ungulate species. Table S5: Standard deviation of heterozygosity of 500-kb windows across autosomal scaffolds larger 1 Mb of the addax and six other wild ungulate species. Table S7: Results from ROHan analysis using default parameters for the addax (*Addax nasomaculatus*). Table S8: Results from ROHan analysis using default parameters for the scimitar-horned oryx (*Oryx dammah*). Text S2: ZMB MAM 2165 Skin Sample. Table S9: Pooling strategy for hybridization capture batch 1. Table S10: Pooling strategy for hybridization capture batch 2. Table S11: Paired-end sequencing yields of four historical and one contemporary addax samples from paired-end sequencing mapped to the addax mitochondrial genome. Table S12: Single-end sequencing yields of seven historical addax samples mapped to the addax mitochondrial genome. Table S13: Mitochondrial sequences from Genbank included in the calibrated Bayesian species phylogeny. Text S3: Fossil Calibrations for the Bayesian Species Phylogeny. Table S14: Calculated mutation rates per year and per generation time assuming different splits of *Oryx*/*Addax* lineage from the species phylogeny assuming a generation time of 6.7 years. Text S4: Mitochondrial Genomes of the Sable Antelope (*Hippotragus niger*). Table S15: Sequences used for the mitochondrial diversity comparison using pairwise distances. Table S16: Average pairwise distances (k) of seven complete mitochondrial genomes per species for the addax and seven wild ungulate species. Supplementary File S2: Supplementary Table S6: Scaffold IDs that aligned to the X chromosome of the domestic goat, the Y chromosome of the wild goat, and the mitochondrial genome of the respective species using SatsumaSynteny v2.0. Supplementary File S3: Mitochondrial species alignment used to build the calibrated Bayesian species phylogeny. Supplementary File S4: Mitochondrial population alignment used to build the Bayesian population phylogeny. Supplementary File S5: Mitochondrial population alignment used to build the phylogenetic network.
