# Supplementary material for: Diversity and Paleodemography of the Addax (Addax nasomaculatus), a Saharan Antelope on the Verge of Extinction
Source: Genes (Basel). 2021 Aug 11;12(8):1236. doi: 10.3390/genes12081236 (PMC8394336; doi:10.3390/genes12081236)
Supplement: Supplementary file 1 [file genes-12-01236-s001.zip › Supplementary_File_S1.pdf]

Diversity and Paleodemography of the Addax (*Addax nasomaculatus*), a Saharan Antelope on the Verge of Extinction

Elisabeth Hempel, Michael V. Westbury, José H. Grau, Alexandra Trinks, Johanna L.A. Paijmans, Sergei Kliver, Axel Barlow, Frieder Mayer, Johannes Müller, Lei Chen, Klaus-Peter Koepfli, Michael Hofreiter and Faysal Bibi

Supplement

Nuclear Genome

Sequencing Yields

Table S1. Paired-end sequencing yields from our contemporary addax sample (IZW 607/10) mapped to the addax nuclear assembly (this study). Two sequencing runs were combined before processing. Merged and unmerged reads were used.

Sample ID	Run # (SRR #)	Total reads	Total read pairs	Pairs <30 bp	reads for mapping	# mapped reads	# uniquely mapped reads	# mapped bp
IZW 607/10	2 (SRR15193276)	443,054,914	221,527,457					
	3 (SRR15193277)	1,015,792,906	507,896,453					
	**total**	1,458,847,820	729,423,910	379,657	1,458,088,506	785,232,193	721,513,267	122,974,874,650

Table S2. Paired-end sequencing yields from our contemporary addax sample (IZW 607/10) mapped to the scimitar-horned oryx nuclear assembly (https://www.dropbox.com/s/ake2hy73998pji8/oryx.1_HiC.fasta.gz?dl=0 [1]). Two sequencing runs were combined before processing. Merged and unmerged reads were used.

Sample ID	Run # (SRR #)	Total reads	Total read pairs	Pairs <30 bp	reads for mapping	# mapped reads	# uniquely mapped reads	# mapped bp
IZW 607/10	2 (SRR15193276)	443,054,914	221,527,457					
	3 (SRR15193277)	1,015,792,906	507,896,453					
	**total**	1,458,847,820	729,423,910	379,657	1,458,088,506	819,553,292	749,383,652	128,967,723,128

Text S1. Assembly Statistics

The three sequencing runs resulted in a total of 1,458,847,820 150 bp paired-end reads (729,423,910 read pairs) and 108,306,216 75 bp paired-end reads (54,153,108 read pairs). According to k-mer distribution, the total genome size was estimated around 3.05 Gbp with a 45.69x coverage by KrATER (https://github.com/mahajrod/KrATER). A total of 6.35 billion distinct k-mers with an estimated 0.6% of error containing k-mers were counted in Jellyfish2 [2]. However, calculating the coverage by taking the ratio of mapped base pairs to genome size from mapping the raw reads of the addax to its new assembly suggests 44.00x.

PSMC

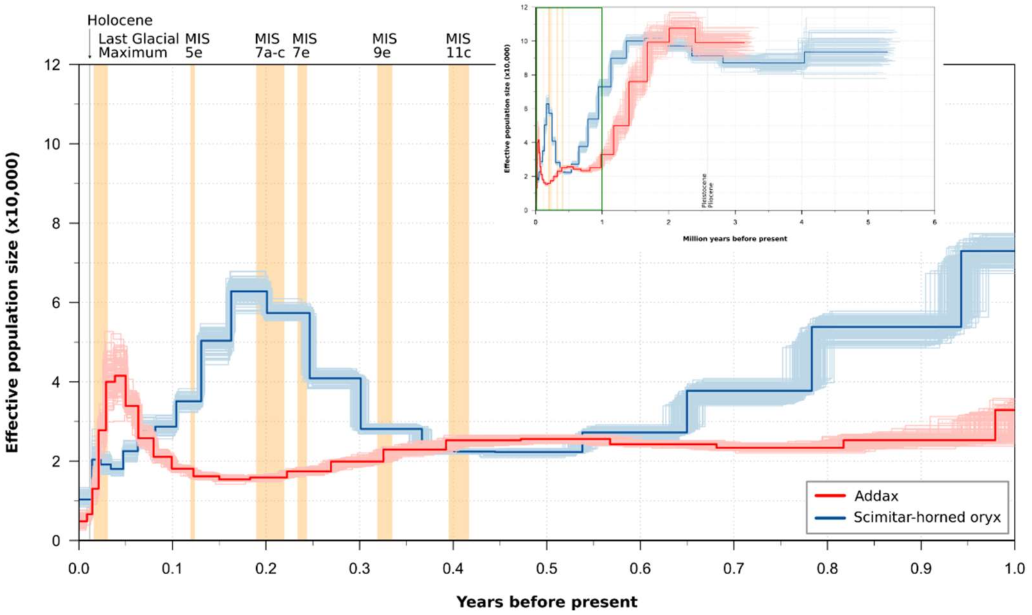

Figure S1. Pairwise sequential Markovian coalescent model for the addax and the scimitar-horned oryx on a linear scale. Changes in effective population size N_e (with 100 bootstrap repetitions) in addax and scimitar-horned oryx based on an autosomal pairwise sequential Markovian coalescent model on a linear scale of one million years and six million years (insert) (addax: generation time 6.8 years, mutation rate per generation (μ) 1.09 x 10^{-8} [3], this study; scimitar-horned oryx: generation time 6.2 years, mutation rate per generation (μ) 9.95 x 10^{-9} [4], this study). Last Glacial Maximum and Marine Isotope Stages (MIS) representing interglacial periods of the last 500,000 years are marked in light orange. Scimitar-horned oryx data from Humble et al. [1].

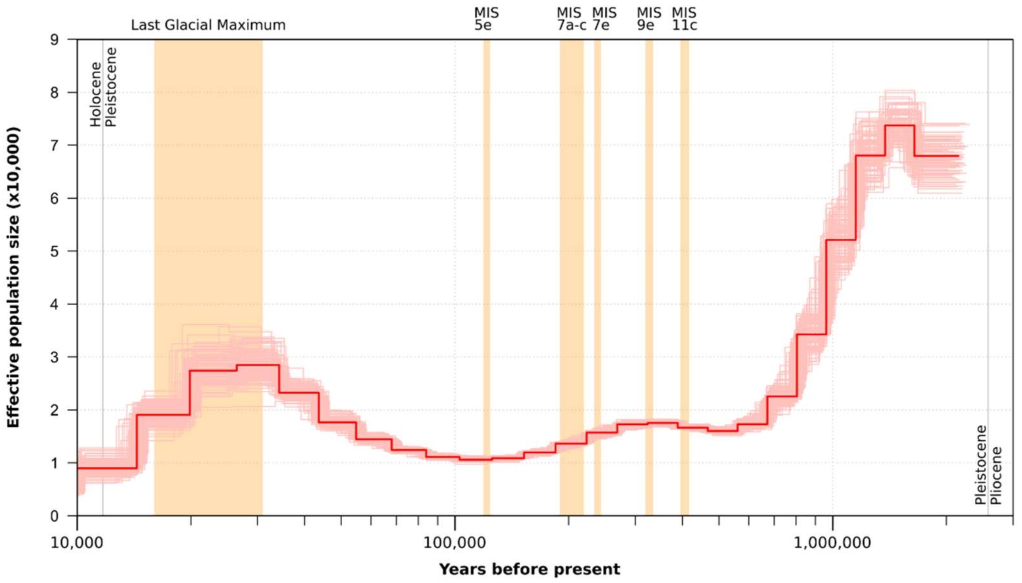

Figure S2. Pairwise sequential Markovian coalescent model for the addax with a mutation rate per generation of 1.59 x 10^{-8}. Changes in effective population size N_e (with 100 bootstrap repetitions) of the addax over years before present based on an autosomal pairwise sequential Markovian coalescent model (generation time 6.8 years, mutation rate per generation (μ) 1.59 x 10^{-8} [3], this study). Last Glacial Maximum and Marine Isotope Stages (MIS) representing interglacial periods of the last 500,000 years are marked in light orange.

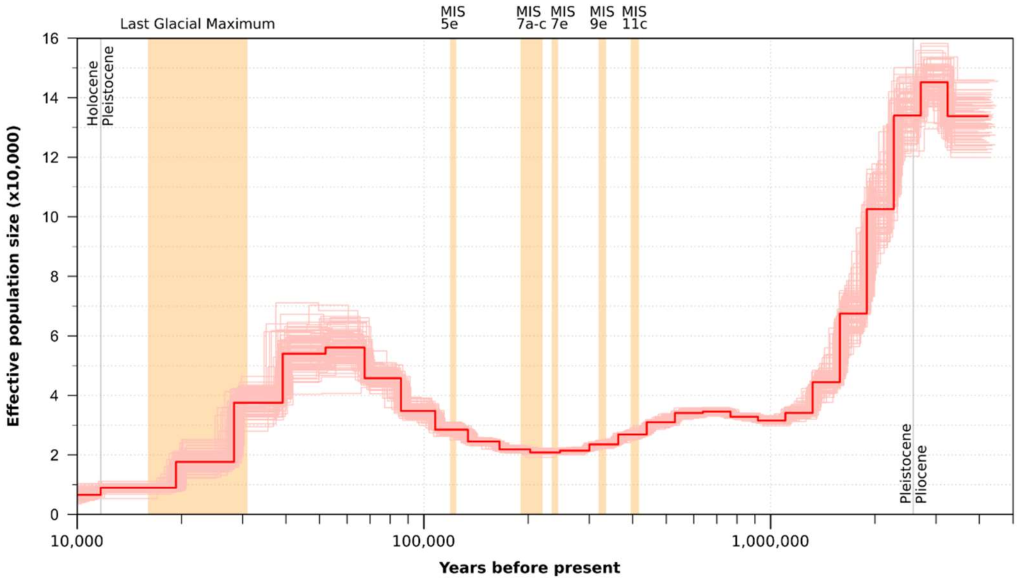

Figure S3. Pairwise sequential Markovian coalescent model for the addax with a mutation rate per generation of 8.08 x 10^{-9}. Changes in effective population size N_e (with 100 bootstrap repetitions) of the addax over years before present based on an autosomal pairwise sequential Markovian coalescent model (generation time 6.8 years, mutation rate per generation (μ) 8.08 x 10^{-9} [3], this study). Last Glacial Maximum and Marine Isotope Stages (MIS) representing interglacial periods of the last 500,000 years are marked in light orange.

Heterozygosity Estimate

Table S3. Accession numbers of raw reads and genome assemblies used in the heterozygosity estimate.

Species	SRR number	Assembly	References
Antidorcas marsupialis	SRR6922977–SRR6922982, SRR6922984–SRR6922991	GCA_006408585.1	[5]
Hippotragus niger	SRR8366605–SRR8366607	GCA_006942125.1	[6]
Kobus ellipsiprymnus	SRR6873226, SRR6873228–SRR6873235	GCA_006410655.1	[5]
Oryx dammah	SRR11529474	https://www.dropbox.com/s/ake2hy73998pji8/oryx.1_HiC.fasta.gz?dl=0	[1]
Oryx gazella	SRR7503152–SRR7503154	GCA_003945745.1	[7]
Syncerus caffer	SRR6872647–SRR6872656	GCA_006408785.1	[5]

Table S4. Estimated autosomal heterozygosity for all scaffolds larger 1 Mb and 20–100-Mb windows in ANGSD v0.923 [8] for the addax and six other wild ungulate species.

Scientific name	Autosomal heterozygosity	SD (20-Mbp windows)	SD (50-Mbp windows)	SD (100-Mbp windows)
Addax nasomaculatus	0.0002766165	0.00008189112	0.00005494494	0.00004436772
Antidorcas marsupialis	0.001532147	0.0001218985	0.0001083543	0.00009368998
Hippotragus niger	0.0004754542	0.00005950143	0.00004133836	0.00003353274
Kobus ellipsiprymnus	0.001185057	0.00007399271	0.00007231166	0.00005392196
Oryx dammah	0.0004216303	0.00007043427	0.00004081933	0.00002860088
Oryx gazella	0.0003552609	0.00007158148	0.00006985737	0.00008898895
Syncerus caffer	0.001551208	0.00009111136	0.0000774397	0.00005201608

Table S5. Standard deviation of heterozygosity of 500-kb windows across autosomal scaffolds larger 1 Mb of the addax and six other wild ungulate species.

**Scientific name**	**SD**
Addax nasomaculatus	0.0001655268
Antidorcas marsupialis	0.0003644216
Hippotragus niger	0.0002003281
Kobus ellipsiprymnus	0.0002895792
Oryx dammah	0.000220255
Oryx gazella	0.0002023727
Syncerus caffer	0.0003775935

Table S6. Scaffold IDs that aligned to the X chromosome of the domestic goat (CM001739.2 [9]), the Y chromosome of the wild goat (CM003213.1 [10]), and the mitochondrial genome of the respective species using SatsumaSynteny v2.0 [11].

see Supplementary File S2

Inbreeding

Table S7. Results from ROHan analysis [12] using default parameters for the addax (*Addax nasomaculatus*). Differences between the heterozygosity estimate are due to differences in the programs ROHan and ANGSD [8] and the usage of the option "-baq 1" for the input file for the latter [13]. Differences in ROH are due to the different window sizes used in these programs (see discussion in main text).

	Mean	Minimum	Maximum
**Global heterozygosity rate**	0.00117812	0.00110494	0.00133514
**Heterozygosity in ROH/nonROH**	0.00117898	0.00110896	0.00132781
**Segments unclassified**	0	0	0
**Segments unclassified (%)**	0	0	0
**Segments in ROH**	0	0	0
**Segments in ROH (%)**	0	0	0
**Segments in non-ROH**	2,292,000,000	2,292,000,000	2,292,000,000
**Segments in non-ROH (%)**	100	100	100
**Avg. length of ROH**	0	0	0

Table S8. Results from ROHan analysis [12] using default parameters for the scimitar-horned oryx (*Oryx dammah*). Differences between the heterozygosity estimate are due to differences in the programs ROHan and ANGSD [8] and the usage of the option "-baq 1" for the input file for the latter [13]. Differences in ROH are due to the different window sizes used in these programs (see discussion in main text).

	Mean	Minimum	Maximum
**Global heterozygosity rate**	0.00168771	0.00167712	0.0018941
**Heterozygosity in ROH/nonROH**	0.0015707	0.00149003	0.00178023
**Segments unclassified**	13,000,000	4,000,000	29,000,000
**Segments unclassified (%)**	1.04	0.32	2.32
**Segments in ROH**	112,000,000	15,000,000	131,000,000
**Segments in ROH (%)**	9.05416	1.20385	11.5929
**Segments in non-ROH**	1,125,000,000	1,115,000,000	1,206,000,000
**Segments in non-ROH (%)**	90.9458	89.4864	100
**Avg. length of ROH**	4,666,670	2,500,000	5,458,330

Mitochondrial Genomes

Text S2. ZMB MAM 2165 Skin Sample

From specimen ZMB MAM 2165 we also processed a skin sample in the same way as stated in the methods of the main text. Since this sample proved to be of much lower quality and had ambiguous/missing data and we already obtained a high quality mitochondrial genome from the skull, we did not use this sequence in the analysis. All non-ambiguous positions of the sequence originating from the skin sample were the same as in the sequence originating from the skull sample.

Hybridization Capture

Table S9. Pooling strategy for hybridization capture batch one. Samples were pooled so that each sample had a final concentration of 10 nM in the combined pool, and each blank a final concentration of 2.5 nM (ExBlk = Extraction blank, LiBlk = Library blank).

**Sample**	**Concen-tration [ng/µl]**	**Average fragment size [bp]**	**Estimated molarity [nM]**	**Final concen-tration of pool [nM]**	**Volume of library into pool [µl]**	**Total amount of DNA in pool [ng]**
ZMB MAM 2165 (bone)	22.6	205	167.0362	10	10.0577	227.304
ZMB MAM 7424	22.6	202	169.5170	10	9.9105	223.9776
ZMB MAM 8836	31.8	190	253.5885	10	6.6249	210.672
ZMB MAM 8837	29.6	200	224.2424	10	7.4919	221.76
ZMB MAM 8838	37.2	212	265.8662	10	6.3190	235.0656
ZMB MAM 8839	35	195	271.9503	10	6.1776	216.216
ZMB MAM 8840	23.4	197	179.9723	10	9.3348	218.4336
ExBlk	3.86	164	35.6615	2.5	11.7774	45.4608
LiBlk	3.64	182	30.3030	2.5	13.8600	50.4504

Table S10. Pooling strategy for hybridization capture batch two. Samples were pooled so that each sample had a final concentration of 8 or 4 nM in the combined pool, and each blank a final concentration of 2.5 nM (ExBlk = extraction blank, LiBlk = library blank).

Sample	Concen-tration [ng/µl]	Average fragment size [bp]	Estimated molarity [nM]	Final concen-tration of pool [nM]	Volume of library into pool [µl]	Total amount of DNA in pool [ng]
ZMB MAM 2165 (skin)	35	166	319.45	8	4.21	147.24
ZMB MAM 2166	18.5	195	143.74	8	9.35	172.97
ZMB MAM 2167	21.2	170	188.94	8	7.11	150.79
ZMB MAM 35370 (sample 1)	9.36	183	77.49	8	17.34	162.32
ZMB MAM 35370 (sample 2)	5.46	214	38.65	4	17.38	94.91
ExBlk1	28.6	135	320.98	2.5	1.31	37.42
LiBlk1	16.3	135	182.94	2.5	2.29	27.42
ExBlk2	9.66	167	87.64	2.5	4.79	46.29
ExBlk2	27	156	262.23	2.5	1.60	43.24

Sequencing Yields

Table S11. Paired-end sequencing yields of four historical and one contemporary addax samples from paired-end sequencing mapped to the addax mitochondrial genome (JN632591 [14]). For ZMB MAM 35370 as well as IZW 607/10 two sequencing runs were combined before processing. Only merged reads were used. "shifted" refers to the second mapping to a reference with 400 bp shifted from the end to the beginning of the reference.

Sample ID	Run # (SRR #)	Total reads	Total read pairs	Pairs <30 bp	Reads for mapping	# mapped reads	# uniquely mapped reads	# mapped bp
ZMB MAM 2165 (skin)	1	74,569,914	37,284,957	12,039,150	23,065,928	21,216,731	10,297	439,390
shifted reference					23,065,928	21,160,738	10,284	438,675
ZMB MAM 2166	1 (SRR15177984)	29,700,668	14,850,334	1,293,558	13,254,337	10,873,166	590,929	40,356,217
shifted reference					13,254,337	10,842,617	589,917	40,295,399
ZMB MAMA 2167	1 (SRR15177983)	22,397,296	11,198,648	2,303,305	8,729,820	7,487,493	57,266	2,721,606
shifted reference					8,729,820	7,468,856	57,197	2,718,991
ZMB MAM 35370 (library 1 + 2 combined)	1 & 2 (SRR15177982 & SRR15177981)	9,862,708	4,931,354	343,185	4,485,368	4,059,439	299,906	21,803,679
shifted reference					4,485,368	4,046,214	299,141	21,745,485
IZW 607/10	2 & 3 (SRR15193276 & SRR15193277)	1,458,847,820	729,423,910	379,657	435,690,532	3,360,122	2,086,694	132,335,998
shifted reference					435,690,532	3,355,371	2,081,702	132,464,800

Table S12. Single-end sequencing yields of seven historical addax samples mapped to the addax mitochondrial genome (JN632591 [14]). "shifted" refers to the second mapping to a reference with 400 bp shifted from the end to the beginning of the reference.

Sample ID	Run # (SRR #)	Total reads	Reads <30 bp	Reads for mapping	# mapped reads	# uniquely mapped reads	# mapped bp
ZMB MAM 2165 (bone)	1 (SRR15177987)	5,662,219	535,378	5,126,841	2,815,934	25,519	1,689,186
shifted reference					2,806,804	25,473	1,686,206
ZMB MAM 7424	1 (SRR15177980)	9,117,398	796,378	8,320,911	5,505,163	54,832	3,388,115
shifted reference					5,498,236	54,770	3,384,368
ZMB MAM 8836	1 (SRR15177979)	17,103,221	1,179,131	15,924,090	11,744,228	90,373	5,562,429
shifted reference					11,705,219	90,156	5,549,010
ZMB MAM 8837	1 (SRR15177978)	6,929,504	576,120	6,353,384	3,898,814	37,297	2,442,962
shifted reference					3,886,454	37,201	2,436,557
ZMB MAM 8838	1 (SRR15177977)	4,493,044	282,425	4,210,619	2,483,723	26,298	1,791,495
shifted reference					2,473,911	26,237	1,787,365
ZMB MAM 8839	1 (SRR15177986)	5,878,560	521,186	5,357,374	3,338,068	34,153	2,217,235
shifted reference					3,326,187	34,087	2,213,132
ZMB MAM 8840	1 (SRR15177985)	2,574,869	199,959	2,374,910	1,252,311	15,910	1,048,241
shifted reference					1,250,092	15,911	1,048,435

Bayesian Phylogenies

Table S13. Mitochondrial sequences from GenBank included in the calibrated Bayesian species phylogeny.

**Species**	**Accession number**	**References**
Alcelaphus buselaphus	JN632593	[14]
Connochaetes gnou	JN632626	[14]
Connochaetes taurinus	JN632627	[14]
Damaliscus lunatus	KF955546	[15]
Damaliscus pygargus phillipsi	FJ207530	[16]
Hippotragus equinus	JN632647	[14]
Hippotragus leucophaeus	MW222233	[17]
Hippotragus niger	JN632648	[14]
Oryx beisa	JN632676	[14]
Oryx dammah	JN632677	[14]
Oryx gazella	JN632678	[14]
Oryx leucoryx	JN632679	[14]
Tragelaphus strepsiceros	NC_020752	[14]

Text S3. Fossil Calibrations

The stem Hippotragini, crown Alcelaphini, crown Hippotragini and crown Bovidae justifications for calibrations follow Bibi [18] and can be found there. Here only the input numbers for priors in BEAUTi v2.5.0 [19,20] are stated.

Clade Name

Definition: phylogenetic definition of the clade in question

Calibration point: name and placing of the calibration point

Minimum age: numerical age of the oldest known fossil

Probability distribution type, 5–95% probability range in Myr, calculated by multiplying minimum age by 1.25 (M & S used to create the required distribution range for median and mean in BEAUTi v2.5.0).

1. museum ID of fossil specimen(s)
2. source(s) for morphological and phylogenetic descriptions of the relevant specimen
3. clade monophyly and possible morphological and molecular phylogenetic analyses disagreements
4. locality and stratigraphic information of the specimen
5. numeric age for the specimen

Crown Hippotragini

Definition: This clade originates with the last common ancestor of *Hippotragus equinus*, *Hippotragus niger*, *Hippotragus leucophaeus*, *Oryx gazella*, *Oryx beisa*, *Oryx dammah* and *Oryx leucoryx*.

Calibration point: crown Hippotragini

Minimum age: 3.6 Ma

Log Normal 3.65–4.5 Ma (offset=3.6, M=0.3, S=0.9, mean=3.90, median=3.80)

See Bibi [18] for further explanations.

Stem Hippotragini

Definition: The clade consists of all Hippotragini, living or extinct.

Calibration point: stem Hippotragini

Minimum age: 6.4 Ma

Log Normal 6.44–8.07 Ma (offset=6.4, M=0.5, S=1.1, mean=6.9, median=8.07)

See Bibi [18] for further explanations.

Crown Connochaetes

Definition: This clade originates with the last common ancestor of *Connochaetes taurinus* and *Connochaetes gnou*.

Calibration point: node uniting *Connochaetes gnou* and *Connochaetes taurinus*

Minimum age: 1.0 Ma

Log Normal 1.02–1.25 Ma (offset=1.0, M=0.1, S=0.7, median=1.08, mean=1.25)

1. *Connochaetes gnou* from Cornelia (see Brink [21,22]).
2. Apomorphies of the Cornelia specimen in Brink [21,22].
3. Morphological and molecular analyses agree that *Connochaetes* is monophyletic (e.g. Vrba [23], Arctander et al. [24], Hassanin et al. [14]).
4. see Brink [22]
5. "Brink's [21,22] studies of fossil wildebeest material from Cornelia-Uitzoek (~1–0.7 Ma) in South Africa suggests that the differentiation of the *C. gnou* lineage from an ancestral (*C. taurinus* like) morphology occurred around this time. This suggests a younger (~1 Ma) minimum age for the divergence of the blue and black wildebeests than previously proposed [23]. Short mitochondrial branch lengths between *C. taurinus* and *C. gnou* [14,15,18] also support a recent divergence scenario." (from Bibi et al. [25]).

Vrba [23] demonstrated that *C. gentryi* was on the stem lineage of the *Connochaetes* clade, and this was confirmed by Bibi et al. [25]. *C. gentryi* is known from as young as 1.9 Ma (Upper Burgi Member at Koobi For a; Harris [26]). It might be 1.6 Ma or younger if *C. gentryi* and

C. *africanus* from Bed II (~1.7–1.2 Ma) at Olduvai Gorge [27] are accepted to be synonyms (see Bibi et al. [25]). Bibi et al. [25] and Brink [21] did not agree with Gentry & Gentry [28] and Vrba [23] that *C*. *africanus* represents an early representative on the *C*. *gnou* lineage.

The maximum age of the *Connochaetes* crown clade is therefore unlikely to be older than 1.9 Ma or 1.6 Ma.

Crown Alcelaphini

Definition: This clade originates with the last common ancestor of *Alcelaphus buselaphus*, *Damaliscus pygargus*, *Damaliscus lunatus*, *Connochaetes taurinus* and *Connochaetes gnou*.

Calibration point: crown Alcelaphini

Minimum age: 4.5 Ma

Log Normal 4.51–5.63 Ma (offset=4.5, M=0.3, S=1.4, mean=4.8, median=4.61)

See Bibi [18] for further explanations.

Crown Bovidae

Definition: This clade originates with the last common ancestor of *Bos taurus* and *Gazella dorcas*.

Calibration point: crown Bovidae

Age: 18.0 Ma

Normal 16.0–20.0 Ma (Mean=18.0, Sigma=1.2, median=18.0)

See Bibi [18] for further explanations.

Table S14. Calculated mutation rates per year and per generation time assuming different splits of *Oryx*/*Addax* lineage from the species phylogeny assuming a generation time of 6.7 years [3].

	**Mean**	**95% interval min**	**95% interval max**
**Estimated split *Oryx*/*Addax* lineages**	2.2103 Mya BP	1.515 Mya BP	2.9833 Mya BP
**Mutation rate per year**	1.626×10^{-9}	2.37×10^{-9}	1.20×10^{-9}
**Mutation rate per generation**	1.089×10^{-8}	1.59×10^{-8}	8.07×10^{-9}

Text S4. Mitochondrial Genomes of the Sable Antelope (*Hippotragus niger*)

Mitochondrial genomes for *Hippotragus niger* were generated using SRR8366677–SRR8366681 and SRR8366605–SRR8366607 from GenBank [6]. Sequencing runs for the same specimens were combined before processing. Illumina adapters were removed (overlap 1 bp) with Cutadapt v2.8 [29] and reads shorter than 30 bp removed. Subsequently, overlapping reads were merged with a maximum overlap of 101 bp in FLASH v1.2.11 [30]. Mapping was conducted with BWA v.0.7.17 with the aln algorithm [31] and default settings. As reference the mitochondrial genome JN632648 of *H. niger* was used [14]. All reads were filtered with SAMtools view v1.10 [32] for a mapping quality of 30, followed by a duplicate removal step with Picard MarkDuplicates v2.22.0 (Picard Toolkit 2020 - http://broadinstitute.github.io/picard). The bam file of sample SB134 was manually edited in position 15,492 in the control region which resulted in an insertion of an A in a poly-A region compared to the reference. For base calling a 85% majority rule threshold was used to generate a consensus sequence with a coverage of 3x and the "trim to reference" option using Geneious R10 v10.2.3 [33] (https://www.geneious.com). To improve the mapping at the ends of the sequences, reads were treated again in the same way as before while using a reference in which 300 bp were shifted from the end to the beginning of the reference. Again, only the bam file of sample SB134 was manually edited before calling the consensus sequence. After generating the consensus sequences, the part of the sequence corresponding to the shifted 300 bp was moved to the end of the sequence. Subsequently, from the two generated

consensus sequences per sample one consensus sequence using a 50% majority rule threshold for base calling was generated (option "50% - Strict: Bases matching at least 50% of the sequences"). This resulted in improved sequences for SB381, SB2027 and SB1954.

Table S15. Sequences used for the mitochondrial diversity comparison using pairwise distances.

Species	Sequences used in comparison	References
Addax nasomaculatus	JN632591, MZ474958 (ZMB MAM 35370), MZ474955 (ZMB MAM 2165), MZ474961 (ZMB MAM 8837), MZ474963 (ZMB MAM 8839), MZ474964 (ZMB MAM 8840), MZ474965 (IZW 607/10)	[14], this study
Alces alces	MK644899, MK644908, MK644911, MK644925, MK644927 (2x), MK644928	[34]
Bison bison	GU946978, GU946980, GU946982, GU946984, GU946987, GU946990, GU946994	[35]
Bison bonasus	JN632602, KY055664(6x)	[14,36]
Equus przewalskii	JN398403, KT368744, KT368747, KT368749, KT368751, KT368753, KT368755	[37,38]
Hippotragus niger	KM245339, NC_020713, MZ488453 (SB2152), MZ488452 (SB2130), MZ488448 (SB134), MZ488451 (SB2027), MZ488449 (SB381)	[6,14,39], this study
Oryx dammah	JN632677, MT248292, MT248293, MT248294, MT248295, MT248296, MT248297	[1,14]
Syncerus caffer	JQ235528, JQ235532, JQ235533, JQ235535, JQ235537, JQ235539, JQ235541	[40]

Table S16. Average pairwise distances (k) of seven complete mitochondrial genomes per species for the addax and seven wild ungulate species.

Species	Average pairwise distance (k)
Addax nasomaculatus	18
Alces alces	13
Bison bison	0
Bison bonasus	13
Equus przewalskii	50
Hippotragus niger	55
Oryx dammah	54
Syncerus caffer	40

References

1. Humble, E.; Dobrynin, P.; Senn, H.; Chuven, J.; Scott, A.F.; Mohr, D.W.; Dudchenko, O.; Omer, A.D.; Colaric, Z.; Aiden, E.L.; et al. Chromosomal-level genome assembly of the scimitar-horned oryx: Insights into diversity and demography of a species extinct in the wild. *Mol. Ecol. Resour.* **2020**, *20*, 1668–1681, doi:10.1111/1755-0998.13181.
2. Marçais, G.; Kingsford, C. A fast, lock-free approach for efficient parallel counting of occurrences of k-mers. *Bioinformatics* **2011**, *27*, 764–770, doi:10.1093/bioinformatics/btr011.
3. Krause, F. EEP Addax *Addax nasomaculatus - Population Analysis 2020*; Zoo Hannover, Hannover, Germany, 2020.
4. Gilbert, T.; Langenhorst, T. *International Studbook for the Scimitar-Horned Oryx* Oryx dammah; 14th ed.; Marwell Wildlife: Winchester, 2019.
5. Chen, L.; Qiu, Q.; Jiang, Y.; Wang, K.; Lin, Z.; Li, Z.; Bibi, F.; Yang, Y.; Wang, J.; Nie, W.; et al. Large-scale ruminant genome sequencing provides insights into their evolution and distinct traits. *Science* **2019**, *364*, eaav6202, doi:10.1126/science.aav6202.
6. Koepfli, K.-P.; Tamazian, G.; Wildt, D.; Dobrynin, P.; Kim, C.; Frandsen, P.B.; Godinho, R.; Yurchenko, A.A.; Komissarov, A.; Krasheninnikova, K.; et al. Whole genome sequencing and re-sequencing of the sable antelope (*Hippotragus niger*): A resource for monitoring diversity in *ex situ* and *in situ* populations. *G3* **2019**, *9*, 1785–1793, doi:10.1534/g3.119.400084.
7. Farré, M.; Li, Q.; Zhou, Y.; Damas, J.; Chemnick, L.G.; Kim, J.; Ryder, O.A.; Ma, J.; Zhang, G.; Larkin, D.M.; et al. A near-chromosome-scale genome assembly of the gemsbok (*Oryx gazella*): An iconic antelope of the Kalahari Desert. *GigaScience* **2019**, *8*, giy162, doi:10.1093/gigascience/giy162.
8. Korneliussen, T.S.; Albrechtsen, A.; Nielsen, R. ANGSD: Analysis of Next Generation Sequencing Data. *BMC Bioinform.* **2014**, *15*, 356, doi:10.1186/s12859-014-0356-4.
9. Dong, Y.; Xie, M.; Jiang, Y.; Xiao, N.; Du, X.; Zhang, W.; Tosser-Klopp, G.; Wang, J.; Yang, S.; Liang, J.; et al. Sequencing and automated whole-genome optical mapping of the genome of a domestic goat (*Capra hircus*). *Nat. Biotechnol.* **2013**, *31*, 135–141, doi:10.1038/nbt.2478.
10. Dong, Y.; Zhang, X.; Xie, M.; Arefnezhad, B.; Wang, Z.; Wang, W.; Feng, S.; Huang, G.; Guan, R.; Shen, W.; et al. Reference genome of wild goat (*Capra aegagrus*) and sequencing of goat breeds provide insight into genic basis of goat domestication. *BMC Genom.* **2015**, *16*, 431, doi:10.1186/s12864-015-1606-1.
11. Grabherr, M.G.; Russell, P.; Meyer, M.; Mauceli, E.; Alföldi, J.; Di Palma, F.; Lindblad-Toh, K. Genome-wide synteny through highly sensitive sequence alignment: Satsuma. *Bioinformatics* **2010**, *26*, 1145–1151, doi:10.1093/bioinformatics/btq102.
12. Renaud, G.; Hanghøj, K.; Korneliussen, T.S.; Willerslev, E.; Orlando, L. Joint estimates of heterozygosity and runs of homozygosity for modern and ancient samples. *Genetics* **2019**, *212*, 587–614, doi:10.1534/genetics.119.302057.
13. Prasad, A.; Lorenzen, E.D.; Westbury, M.V. Evaluating the role of reference-genome phylogenetic distance on evolutionary inference. *Mol. Ecol. Resour.* **2021**, doi:10.1111/1755-0998.13457.
14. Hassanin, A.; Delsuc, F.; Ropiquet, A.; Hammer, C.; Jansen van Vuuren, B.; Matthee, C.; Ruiz-Garcia, M.; Catzeflis, F.; Areskoug, V.; Nguyen, T.T.; et al. Pattern and timing of diversification of cetartiodactyla (Mammalia, laurasiatheria), as revealed by a comprehensive analysis of mitochondrial genomes. *C. R. Biol.* **2012**, *335*, 32–50, doi:10.1016/j.crvi.2011.11.002.
15. Steiner, C.C.; Charter, S.J.; Houck, M.L.; Ryder, O.A. Molecular phylogeny and chromosomal evolution of Alcelaphini (Antilopinae). *J. Hered.* **2014**, *105*, 324–333, doi:10.1093/jhered/esu004.

16. Hassanin, A.; Ropiquet, A.; Couloux, A.; Cruaud, C. Evolution of the mitochondrial genome in mammals living at high altitude: New insights from a study of the tribe Caprini (Bovidae, antilopinae). *J. Mol. Evol.* **2009**, *68*, 293–310, doi:10.1007/s00239-009-9208-7.
17. Hempel, E.; Bibi, F.; Faith, J.T.; Brink, J.S.; Kalthoff, D.C.; Kamminga, P.; Paijmans, J.L.A.; Westbury, M.V.; Hofreiter, M.; Zachos, F.E. Identifying the true number of specimens of the extinct blue antelope (*Hippotragus leucophaeus*). *Sci. Rep.* **2021**, *11*, 2100, doi:10.1038/s41598-020-80142-2.
18. Bibi, F. A multi-calibrated mitochondrial phylogeny of extant Bovidae (Artiodactyla, ruminantia) and the importance of the fossil record to systematics. *BMC Evol. Biol.* **2013**, *13*, 166, doi:10.1186/1471-2148-13-166.
19. Bouckaert, R.; Heled, J.; Kühnert, D.; Vaughan, T.; Wu, C.-H.; Xie, D.; Suchard, M.A.; Rambaut, A.; Drummond, A.J. BEAST 2: A software platform for Bayesian evolutionary analysis. *PLoS Comput. Biol.* **2014**, *10*, e1003537, doi:10.1371/journal.pcbi.1006650.
20. Bouckaert, R.; Vaughan, T.G.; Barido-Sottani, J.; Duchêne, S.; Fourment, M.; Gavryushkina, A.; Heled, J.; Jones, G.; Kühnert, D.; De Maio, N.; et al. BEAST 2.5: An advanced software platform for Bayesian evolutionary analysis. *PLoS Comput. Biol.* **2019**, *15*, e1006650, doi:10.1371/journal.pcbi.1006650.
21. Brink, J.S. Postcranial evidence for the evolution of the black wildebeest, *Connochaetes gnou*: An exploratory study. *Palaeont. afr.* **1993**, *30*, 61–69.
22. Brink, J.S. The Evolution of the Black Wildebeest, *Connochaetes gnou*, and Modern Large Mammal Faunas in Central Southern Africa. PhD Thesis, University of Stellenbosch: Stellenbosch, South Africa, 2005.
23. Vrba, E.S. New fossils of Alcelaphini and caprinae (Bovidae: Mammalia) from Awash, Ethiopia, and phylogenetic analysis of Alcelaphini. *Palaeont. afr.* **1997**, *34*, 127–198.
24. Arctander, P.; Johansen, C.; Coutellec-Vreto, M.-A. Phylogeography of three closely related African bovids (tribe Alcelaphini). *Mol. Biol. Evol.* **1999**, *16*, 1724–1739, doi:10.1093/oxfordjournals.molbev.a026085.
25. Bibi, F.; Rowan, J.; Reed, K. Late Pliocene Bovidae from Ledi-Geraru (Lower Awash Valley, Ethiopia) and their implications for Afar Paleoecology. *J. Vertebr. Paleontol.* **2017**, *37*, e1337639, doi:10.1080/02724634.2017.1337639.
26. Harris, J.M. Family Bovidae. In *Koobi Fora Research Project Volume III*; Harris, J.M., Ed.; Koobi Fora Research Project; Clarendon Press: Oxford, 1991; Vol. 3, pp. 139–320.
27. Hopwood, A.T. New fossil mammals from Olduvai, Tanganyika Territory. *Ann. Mag. Nat. Hist.* **1934**, *14*, 546–550, doi:10.1080/00222933408654928.
28. Gentry, A.W.; Gentry, A. Fossil Bovidae (Mammalia) of Olduvai Gorge, Tanzania, part 1. *Bull. Br. Mus. Nat. Hist. Geol.* **1978**, *29*, 289–446.
29. Martin, M. Cutadapt removes adapter sequences from high-throughput sequencing reads. *EMBnet.journal* **2011**, *17*, 10–12, doi:10.14806/ej.17.1.200.
30. Magoč, T.; Salzberg, S.L. FLASH: Fast length adjustment of short reads to improve genome assemblies. *Bioinformatics* **2011**, *27*, 2957–2963, doi:10.1093/bioinformatics/btr507.
31. Li, H.; Durbin, R. Fast and accurate short read alignment with Burrows–Wheeler transform. *Bioinformatics* **2009**, *25*, 1754–1760, doi:10.1093/bioinformatics/btp324.
32. Li, H.; Handsaker, B.; Wysoker, A.; Fennell, T.; Ruan, J.; Homer, N.; Marth, G.; Abecasis, G.; Durbin, R.; 1000 Genome Project Data Processing Subgroup. The Sequence Alignment/Map format and SAMtools. *Bioinformatics* **2009**, *25*, 2078–2079, doi:10.1093/bioinformatics/btp352.
33. Kearse, M.; Moir, R.; Wilson, A.; Stones-Havas, S.; Cheung, M.; Sturrock, S.; Buxton, S.; Cooper, A.; Markowitz, S.; Duran, C.; et al. Geneious Basic: An integrated and extendable desktop software platform for the organization and analysis of sequence data. *Bioinformatics* **2012**, *28*, 1647–1649, doi:10.1093/bioinformatics/bts199.
34. DeCesare, N.J.; Weckworth, B.V.; Pilgrim, K.L.; Walker, A.B.D.; Bergman, E.J.; Colson, K.E.; Corrigan, R.; Harris, R.B.; Hebblewhite, M.; Jesmer, B.R.; et al. Phylogeography of moose in western North America. *J. Mammal.* **2020**, *101*, 10–23, doi:10.1093/jmammal/gyz163.

35. Douglas, K.C.; Halbert, N.D.; Kolenda, C.; Childers, C.; Hunter, D.L.; Derr, J.N. Complete mitochondrial DNA sequence analysis of *Bison bison* and bison–cattle hybrids: Function and phylogeny. *Mitochondrion* **2011**, *11*, 166–175, doi:10.1016/j.mito.2010.09.005.
36. Węcek, K.; Hartmann, S.; Paijmans, J.L.A.; Taron, U.; Xenikoudakis, G.; Cahill, J.A.; Heintzman, P.D.; Shapiro, B.; Baryshnikov, G.; Bunevich, A.N.; et al. Complex admixture preceded and followed the extinction of wisent in the wild. *Mol. Biol. Evol.* **2017**, *34*, 598–612, doi:10.1093/molbev/msw254.
37. Achilli, A.; Olivieri, A.; Soares, P.; Lancioni, H.; Kashani, B.H.; Perego, U.A.; Nergadze, S.G.; Carossa, V.; Santagostino, M.; Capomaccio, S.; et al. Mitochondrial genomes from modern horses reveal the major haplogroups that underwent domestication. *Proc. Natl. Acad. Sci. U.S.A.* **2012**, *109*, 2449–2454, doi:10.1073/pnas.1111637109.
38. Der Sarkissian, C.; Ermini, L.; Schubert, M.; Yang, M.A.; Librado, P.; Fumagalli, M.; Jónsson, H.; Bar-Gal, G.K.; Albrechtsen, A.; Vieira, F.G.; et al. Evolutionary genomics and conservation of the endangered Przewalski's horse. *Curr. Biol.* **2015**, *25*, 2577–2583, doi:10.1016/j.cub.2015.08.032.
39. Themudo, G.E.; Rufino, A.C.; Campos, P.F. Complete mitochondrial DNA sequence of the endangered giant sable antelope (*Hippotragus niger variani*): Insights into conservation and taxonomy. *Mol. Phylogenet. Evol.* **2015**, *83*, 242–249, doi:10.1016/j.ympev.2014.12.001.
40. Heller, R.; Brüniche-Olsen, A.; Siegismund, H.R. Cape buffalo mitogenomics reveals a Holocene shift in the African human-megafauna dynamics. *Mol. Ecol.* **2012**, *21*, 3947–3959, doi:10.1111/j.1365-294X.2012.05671.x.
